# CCL5 exposure promotes acute and transient megakaryopoiesis and hematopoietic progenitor expansion

**DOI:** 10.1016/j.stemcr.2026.103014

**Published:** 2026-07-16

**Authors:** Maria N. Barrachina, Dong H. Lee, Virginia Camacho, Karen G. Wang, Ethan Walsey, Estelle Carminita, Isabelle C. Becker, Siobhan Branfield, Daniela Freire, Emma Nikols, Emma L. Berdan, Blen Yohannes, Olivera Miladinovic, Julia Tilburg, Joseph E. Italiano, Kellie R. Machlus

**Affiliations:** 1 Vascular Biology Program, Boston Children’s Hospital, Boston, MA, USA; 2 Department of Surgery, Harvard Medical School, Boston, MA, USA; 3 Harvard Chan Bioinformatics Core, Harvard T.H. Chan School of Public Health, Boston, MA, USA

**Keywords:** megakaryocytes, CCL5, inflammation, hematopoiesis, megakaryopoiesis

## Abstract

During inflammation, megakaryocytes (**MKs**) can expand from hematopoietic stem cells, yet the specific driving factors remain unclear. We investigated whether C-C Motif Chemokine Ligand 5 (**CCL5**) influences this process in murine and human iPSC-derived bone marrow organoid (**BMO**) models. Acute CCL5 administration to mice increased hematopoietic stem and progenitor cell (**HSPC**) populations and increased MKs, including the expansion of MHC II-expressing MKs, characteristic of the immune MK subpopulation. Mechanistically, MK progenitors upregulated CCR1 and CCR5, and MKs showed increased phosphorylation of JAK1, SRC, and STAT1-5. Phenotypically, transcriptomic profiling revealed enhanced expression of genes associated with the cell cycle, inflammatory pathways, and mitochondrial metabolism in MKs isolated from CCL5-treated mice, which was functionally supported by increased mitochondrial activity. Similarly, in human BMOs, CCL5 treatment increased MK numbers. Together, these findings implicate CCL5 as a direct mediator of acute megakaryopoiesis and immune MK expansion during inflammation.

## Highlights

• CCL5 acutely drives megakaryopoiesis and transient HSC expansion
• CCL5 increases CCR1 and CCR5 expression in MkPs and HSCs *in vivo*
• CCL5 reprograms MK transcripts and activates JAK/STAT in immune MKs
• CCL5 boosts megakaryopoiesis in human iPSC-derived bone marrow organoids

## Graphical abstract

**Figure:**
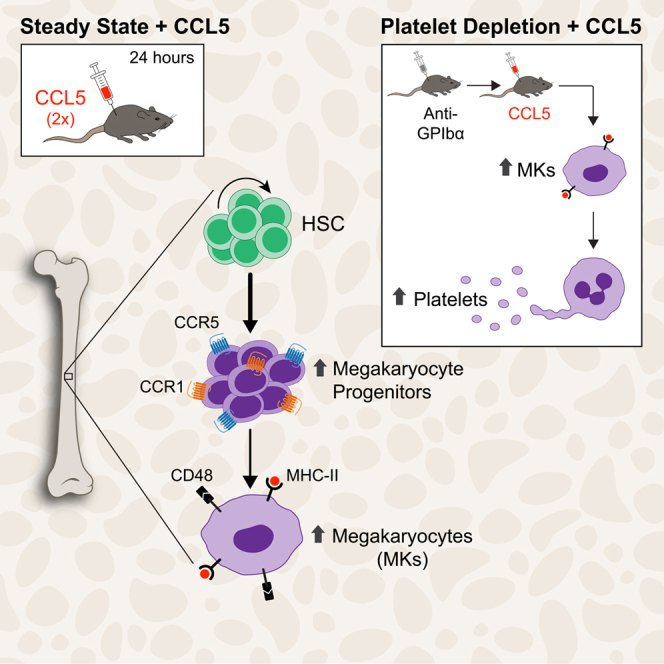

## Teaser

In this article, Barrachina et al. show that the inflammatory chemokine CCL5 acutely expands megakaryocyte progenitors and immune-phenotype megakaryocytes in mice and human bone marrow organoids, accelerates platelet recovery after thrombocytopenia, and reprograms megakaryocyte transcriptional and metabolic states, revealing CCL5 as a conserved driver of emergency megakaryopoiesis.

## Introduction

Traditionally, megakaryopoiesis has been described as a stepwise progression from hematopoietic stem cells (**HSCs**) to megakaryocytes (**MKs**) along the myeloid branch of hematopoiesis, driven by thrombopoietin (**TPO**) signaling through the MPL receptor (Stone et al., 2022). While the lifespan of bone marrow MKs is not known, HSCs treated with TPO require several days to differentiate and mature into MKs prior to subsequent platelet production. However, platelet counts can increase within hours, revealing that HSCs can rapidly give rise to MKs in response to acute platelet consumption or loss, suggesting a TPO-independent effect (Stone et al., 2022; Couldwell and Machlus, 2019; Machlus and Italiano, 2013). In recent years, studies have demonstrated that MKs can also be generated through non-canonical pathways, bypassing the conventional multipotent or bipotent progenitor stages and differentiating more directly from HSCs to MKs (Sanjuan-Pla et al., 2013; Rodriguez-Fraticelli et al., 2018). These alternative differentiation pathways are now recognized as part of megakaryopoiesis and an important part of the hematopoietic system’s ability to rapidly adapt to stress, injury, and inflammation. However, these findings raise important questions regarding why HSCs or multipotent progenitors (**MPPs**) sometimes differentiate through stepwise, lineage-restricted intermediates despite the ability to directly commit to the MK lineage, and which molecular cues govern this decision (Li et al., 2024). Addressing these questions represents a major knowledge gap in MK biology.

Inflammation can have broad effects on hematopoiesis, reprogramming hematopoietic stem and progenitor cells (**HSPCs**) and altering cell fate and function (Caiado et al., 2021; Pietras, 2017). Inflammatory signals can also have more targeted effects and reprogram HSPCs to skew differentiation toward the megakaryocytic lineage to meet urgent platelet demands. Haas and colleagues discovered an interferon I-induced MK-biased HSC subpopulation that expands upon inflammatory stress to efficiently replenish platelets, thus identifying a functional MK-biased HSC population (Haas et al., 2015). Other inflammatory mediators such as interleukin (**IL**)-1β, IL-1α, and IL-6 also enhance MK maturation and platelet production through both direct and indirect mechanisms (Nishimura et al., 2015; Ho et al., 2019).

Interferons and ILs are not the only inflammatory signals capable of influencing hematopoiesis. Chemokines also play important roles in regulating hematopoiesis and maintenance of the hematopoietic niche since they modulate HSC activity and mobilization to alter lineage fate and accelerate differentiation in response to acute physiological demands (Mukaida et al., 2017). Among others, the C-C motif chemokine ligand 5 (**CCL5**) has emerged as a potential modulator of HSC activity. CCL5 levels increase in the bone marrow microenvironment with aging and upon inflammation, two conditions associated with a bias toward myeloid cell production (Ergen et al., 2012; Machlus et al., 2016). In aging, elevated CCL5 levels have been linked to myeloid-biased differentiation (Ergen et al., 2012), while CCL5-deficient mice display reduced myeloid-biased HSCs accompanied by an expanded T cell compartment (Ergen et al., 2012). These findings indicate that age-associated alterations in hematopoietic function may be driven, at least in part, by increased CCL5 expression within the bone marrow (Ergen et al., 2012), though its precise role and mechanisms of action remain incompletely defined. CCL5 administration after total body ionizing radiation treatment in mice accelerates HSPC regeneration and improves HSPC survival (Piryani et al., 2019). Likewise, the deletion of the CCL5 receptor, *Ccr5,* in hematopoietic cells, but not stromal/endothelial cells, delays HSPC expansion and recovery. Intriguingly, a recent study showed that CCR5 marks a subset of HSCs that are myeloid-primed and expand with age (Yılmaz et al., 2026). Ultimately, it has been suggested that the CCL5-CCR5 signaling axis promotes hematopoietic cell cycling and survival, revealing CCL5-CCR5 as a regulator of hematopoietic regeneration and a potential therapeutic target to shorten myelosuppression (Piryani et al., 2019).

While these studies suggest a role for CCL5 in promoting myeloid-biased hematopoiesis during acute and chronic inflammation and aging, the effect of CCL5 on MK differentiation and maturation *in vivo* has not been explored. Further, while inflammation in general can trigger emergency megakaryopoiesis, few factors that acutely expand the MK population have been identified (Haas et al., 2015). Thus, an improved understanding of pathways capable of promoting emergency megakaryopoiesis or transiently expanding MK numbers would advance our fundamental understanding of how MKs are regulated *in vivo*.

Our group previously demonstrated that CCL5 enhanced MK maturation and proplatelet formation via the CCR5-CCL5 axis *in vitro* (Machlus et al., 2016). Here, we sought to extend these findings to the *in vivo* impact of CCL5 on MKs and the preceding hematopoietic differentiation pathways. Additionally, we examined the effects of CCL5 under stress conditions, particularly during platelet recovery following anti-GPIbα-induced platelet depletion in mice. We found that 24-h CCL5 exposure in mice enhanced megakaryopoiesis, characterized by an increased number of MKs and an expansion of MHC II^+^ and CD48^+^ MK populations. Further, CCL5 treatment resulted in increased MK progenitors; these progenitors also upregulated the CCL5 receptors CCR1 and CCR5. Notably, the enhanced megakaryopoiesis seen in mice was mirrored in an iPSC-derived human bone marrow organoid (**BMO**), where CCL5 administration similarly increased MK numbers. In summary, our data reveal CCL5 as a novel, conserved regulator of emergency megakaryopoiesis, coupling inflammatory cues to MK progenitor activation and immune MK expansion across murine and human model systems.

## Results

### Acute CCL5 administration increases bone marrow megakaryocytes

**Figure 1:**
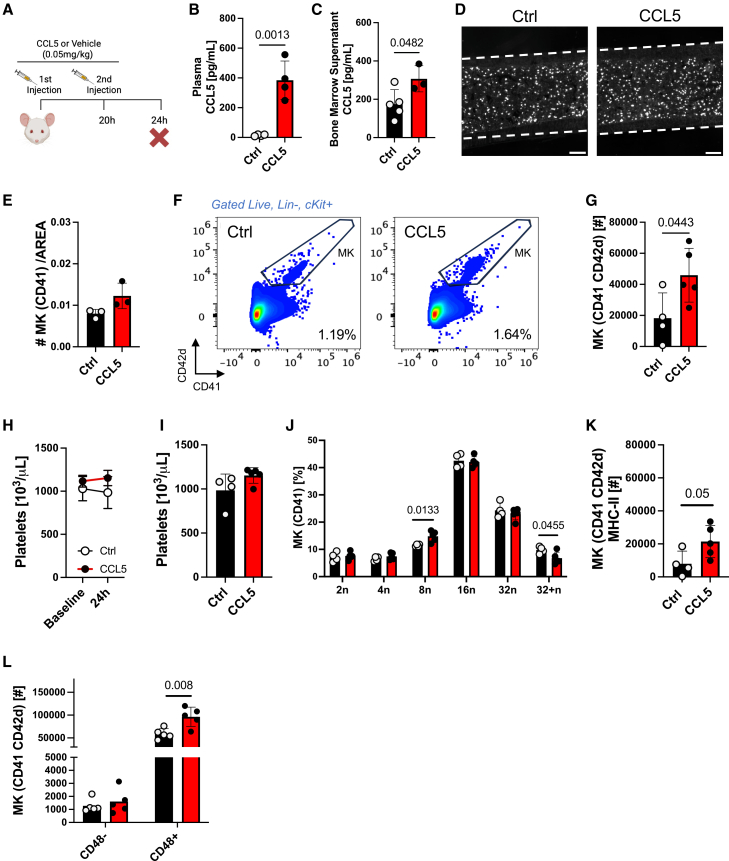
CCL5 enhances megakaryopoiesis *in vivo* (A) Schematic of CCL5 *in vivo* administration protocol. Male mice received retroorbital injections of CCL5 (**CCL5**, 0.05 mg/kg) or vehicle (**Ctrl**) 24- and 4-h prior to euthanasia. (B and C) Quantification of CCL5 levels in (B) plasma and (C) bone marrow supernatant at 24 h by ELISA (*n* = 3–5 mice per condition). (D and E) Femoral cryosections from Ctrl and CCL5-treated mice were stained for CD41. (D) Representative bone marrow images showing MKs (CD41, gray). Scale bars, 200 μm. (E) Quantification of MKs (CD41^+^) per area using ImageJ (*n* = 3). (F–G) (F) Representative plot and (G) quantification of MKs by flow cytometry showing absolute counts following vehicle or CCL5 administration (*n* = 4–5). (H and I) Platelet counts before and after administration of vehicle or CCL5, analyzed using an automated hematology analyzer, Sysmex (*n* = 4–5). (J) Ploidy distribution of native bone marrow MKs assessed by propidium iodide staining and quantified by flow cytometry (*n* = 4). (K) Absolute counts of MKs expressing MHC II, quantified by flow cytometry (*n* = 5). Data are presented as mean ± SD. Statistical analyses were performed using an unpaired, two-tailed Student’s *t* test. *N* represents an individual mouse; experiments were repeated 2–3 times. Illustrations were created by Biorender. *CCL5, C-C motif chemokine ligand 5; MKs, megakaryocytes.*

Our group previously demonstrated that CCL5 enhanced MK maturation and proplatelet formation *in vitro*, suggesting that the chemokine may act as a modulator of megakaryopoiesis (Machlus et al., 2016). To extend these findings, we investigated how CCL5 influences megakaryopoiesis *in vivo*. Mice were treated with 2 doses of 0.05 mg/kg CCL5 or vehicle, and bone marrow and blood counts were assessed after 24 h (Figure 1A). We chose a two-dose regimen (24 h and 4 h before analysis) to maintain elevated CCL5 levels across a window of 24 h that models acute, inflammatory exposure. To confirm that CCL5 levels were elevated, we measured the concentration in plasma and bone marrow supernatant by ELISA after 24 h and observed a significant increase in both compartments in CCL5-treated mice compared to vehicle-treated controls (Figures 1B and 1C). We next examined the MK population in the bone marrow following CCL5 administration by immunofluorescence staining of femoral cryosections, which revealed increased MKs in the CCL5-treated group (Figures 1D and 1E). This was further validated by flow cytometry, demonstrating a significant increase in MKs (Lin^−^CD41^+^CD42d^+^) in CCL5-treated mice compared to controls (Figures 1F and 1G).

We did not observe significant differences in platelet counts, mean platelet volume, or other blood cell counts 24 h after CCL5 treatment (Figures 1H, 1I, and S1A–S1C). Similarly, platelet activation at baseline (resting) or upon stimulation was unaffected by CCL5 administration, as assessed by P-selectin and activated integrin αIIbβ3 exposure (Figures S1D and S1E). Notably, the MK population isolated from CCL5-treated mice was shifted toward low-ploidy cells (*n* = 8), with a concomitant reduction in higher-ploidy MKs (32+ *n*) compared to the vehicle group (Figure 1J). This may explain the absence of changes in platelet numbers, as lower ploidy has been associated with “immune” MK subsets (Sun et al., 2021; Li et al., 2024), which are distinct from platelet-producing MKs (Guo et al., 2024; Sun et al., 2021). Consistent with this finding, CCL5 treatment increased the number of MKs expressing MHC II, a marker characteristic of immune MKs (Figure 1K) (Li et al., 2024; Camacho et al., 2025). Additionally, the proportion of MKs that were CD48^+^ significantly increased following CCL5 administration (Figure 1L). Collectively, these data demonstrate that CCL5 acutely increased MK counts within 24 h of administration and highlight a specific increase in an immune-like MK subset.

### CCL5 selectively increases HSPC numbers and proliferation

**Figure 2:**
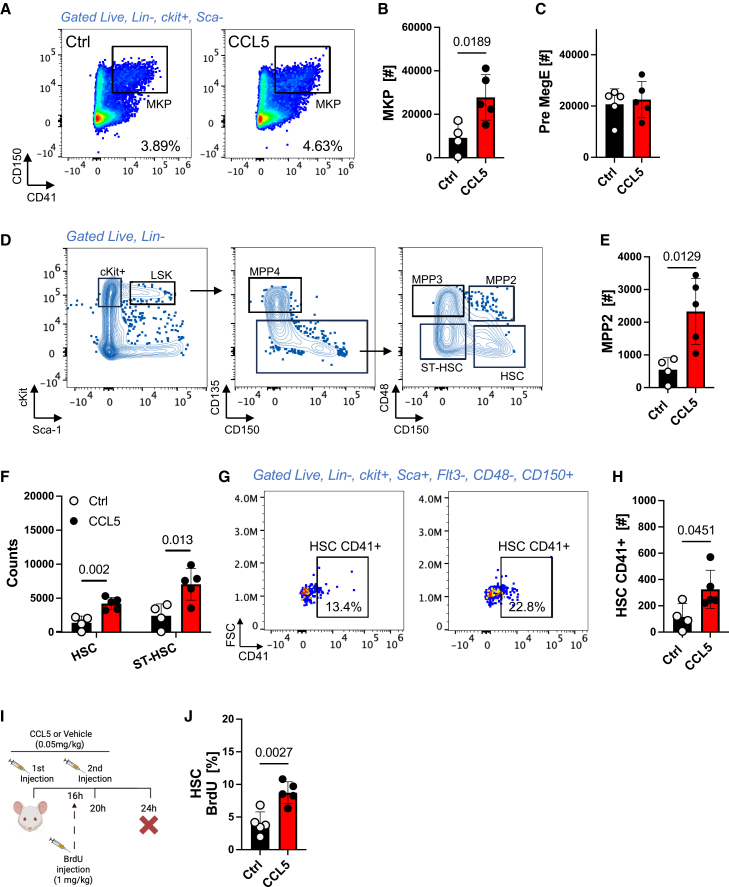
CCL5 stimulation promotes the expansion of MK progenitors (A and B) MkPs were quantified by flow cytometry (*n* = 4–5). (A) Representative plot of MkPs in Ctrl and CCL5-treated mice. (B) Absolute counts of MkPs. (C) Absolute counts of Pre MegEs quantified by flow cytometry (*n* = 4–5). (D) Representative gating strategy to identify HSPCs and multipotent progenitors. (E and F) Absolute counts of (E) MPP2 and (F) HSCs and ST-HSCs quantified by flow cytometry. (G) Representative plot of CD41^+^ HSCs in Ctrl and CCL5-treated mice. (H) Absolute counts of CD41^+^ HSCs. (I) Experimental schematic: BrdU (1 mg/mouse) was administered intraperitoneally 16 h prior to euthanasia and collection, and CCL5 or vehicle was administered 24 and 4 h prior to euthanasia and collection. (J and K) Frequency of BrdU^+^ HSCs, and (K) BrdU^+^ CD41^+^ HSCs (*n* = 5). Data are presented as mean ± SD. Statistical analyses were performed using an unpaired, two-tailed Student’s *t* test. *N* represents an individual mouse and experiments were repeated 2–3 times. Illustrations were created by Biorender. *MkP, megakaryocyte progenitor; MegE, megakaryocyte-erythroid progenitor; HSPC, hematopoietic stem and progenitor cell; HSC, hematopoietic stem cell; ST-HSC, short-term HSC; MPP2, multipotent progenitor type 2.*

Because we observed increased MKs, we next asked whether this effect also extended to MK progenitors (Lin^−^Sca-1^−^c-Kit^+^CD150^+^CD41^+^) (Figure 2A) (Pronk et al., 2007). MkPs were elevated in CCL5-treated mice compared to controls (Figure 2B), while no differences were observed in pre-MK/erythroid progenitors (**Pre-MegEs**, Figure 2C), granulocyte/monocyte progenitors (**GMPs**; Figure S2B), and pre-granulocyte/monocytes (**Pre GMs**, Figure S2C). MPPs, which represent lineage-biased, multipotent populations derived from HSCs, were gated as previously described (Figure 2D) (Pietras et al., 2015). MPP2 cells (Lin^−^Sca-1^+^c-Kit^+^Flt3^–^CD48^+^CD150^+^), biased toward the MK/erythroid lineage, were significantly increased in the CCL5-treated group (Figure 2E). We observed a similar increase in MPP3 (Lin^−^Sca-1^+^c- Kit^+^Flt3^–^CD48^+^CD150^−^) and MPP4 (Lin^−^Sca-1^+^c-Kit^+^Flt3^+^) cells (Figure S2D). Analysis of HSC subsets revealed that CCL5 stimulation increased both HSCs (Lin^−^Sca-1^+^cKit^+^Flt3^–^ CD150^+^CD48^−^) and short-term HSCs (**ST-HSC**, Lin^−^Sca-1^−^c-Kit^+^Flt3^–^CD150^−^CD48^−^; Figure 2F). Notably, CCL5 also significantly expanded the MK-biased HSC population (Lin^−^Sca-1^+^cKit^+^Flt3^–^ CD150^+^CD48^−^CD41^+^) in the bone marrow (Figures 2G and 2H).

Given that CCL5 has been reported to promote proliferation in cancer cells (Gao et al., 2016), we next assessed whether the observed increases in HSCs, particularly the MK-biased HSCs, were associated with enhanced cell proliferation (Figure 2I). BrdU incorporation assays revealed that CCL5-treated mice exhibited increased proliferation in HSCs compared with vehicle controls (Figure 2J). Together, these results reveal that CCL5 significantly increased HSC proliferation and acutely increased both MK progenitor cells and MK-biased HSCs.

### Acute and non-persistent effects of CCL5 on megakaryopoiesis

**Figure 3:**
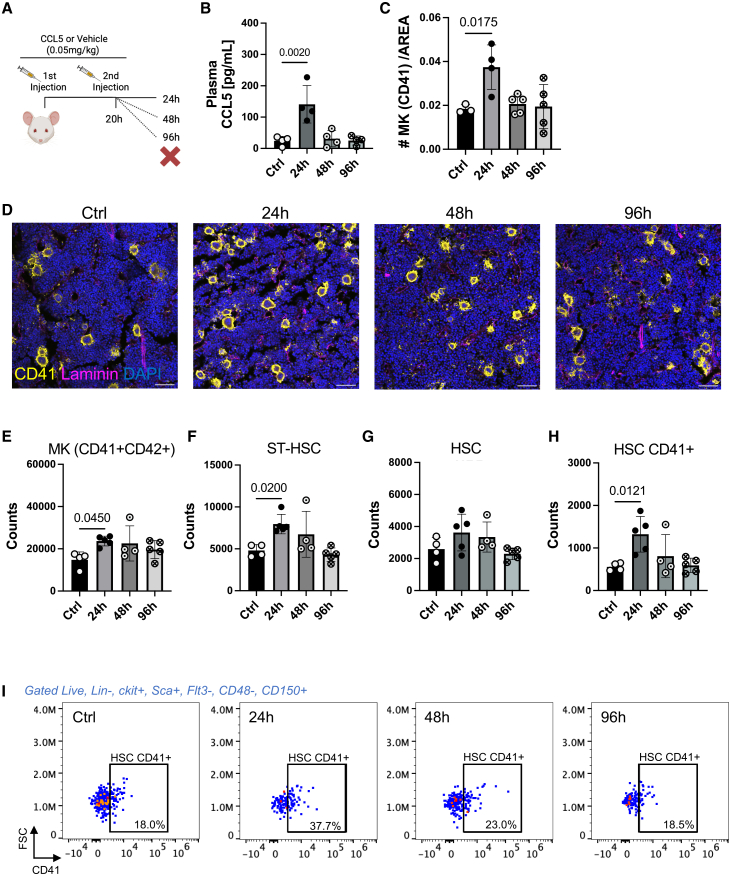
CCL5 induces an acute and transient expansion of megakaryopoiesis (A) Mice received retro-orbital injections of CCL5 (0.5 mg/kg) or vehicle (Ctrl) twice over 24 hours-at hour 0 and 20. Mice were euthanized 24, 48, and 96 h after the first injection; all groups were euthanized simultaneously. (B) Quantification of CCL5 levels in plasma at 24, 48, and 96 h by ELISA (*n* = 4–5). (C and D) Femoral cryosections from Ctrl and CCL5-treated mice stained for CD41. (C) Quantification of MKs (CD41^+^) per area using ImageJ (*n* = 3–5). (D) Representative bone marrow images showing MKs (CD41^+^, yellow), vessels (laminin, pink), and nuclei (DAPI, blue). Scale bars, 100 μm. (E–H) Absolute numbers of (E) MKs, (F) ST-HSCs, (G) HSCs, and (H) CD41^+^ HSCs were quantified by flow cytometry (*n* = 4–5). (I) Representative flow cytometry plot shows CD41^+^ HSCs after CCL5 administration over 24, 48, and 96 h. Data are presented as mean ± SD. Statistical analyses were performed using one-way ANOVA with Dunnett’s multiple comparisons test. *N* represents an individual mouse; experiments were repeated 2 times. Illustrations were created by Biorender. *MKs, megakaryocytes; HSC, hematopoietic stem cell; ST-HSC, short-term HSC.*

Next, we determined the kinetics of CCL5 treatment on different bone marrow populations. Mice were treated as in Figure 1A and then euthanized after 24, 48, and 96h (Figure 3A). Measurement of plasma revealed a marked decrease in CCL5 levels at 48 and 96h (Figure 3B). In addition, while MK levels were elevated 24h following CCL5 administration, MK number declined after 48 and 96h (Figures 3C and 3D). Similar results were obtained by flow cytometry, where MK numbers were significantly increased at 24h and then returned to baseline (Figure 3E) by 48h. Consistent with our previous findings, peripheral blood cell counts were not altered by CCL5 administration over time (Figures S3A–S3D).

We examined the HSPC compartment, focusing on ST-HSCs, HSCs, and MK-biased HSCs (CD41^+^), which expanded following acute CCL5 administration (Figure 2I). Kinetic analysis revealed that the ST-HSC population peaked at 24 h but returned to baseline at 48 and 96 h (Figure 3F), while there were no significant changes in HSCs (Figure 3G). The MK-biased HSC population also showed a significant increase after 24 h (Figures 3H and 3I). These findings demonstrate that the effect of CCL5 on HSPCs and MKs peaks after 24 h, suggesting that CCL5 acts as a rapid stress signal.

### Impact of CCL5 on megakaryopoiesis after platelet depletion

**Figure 4:**
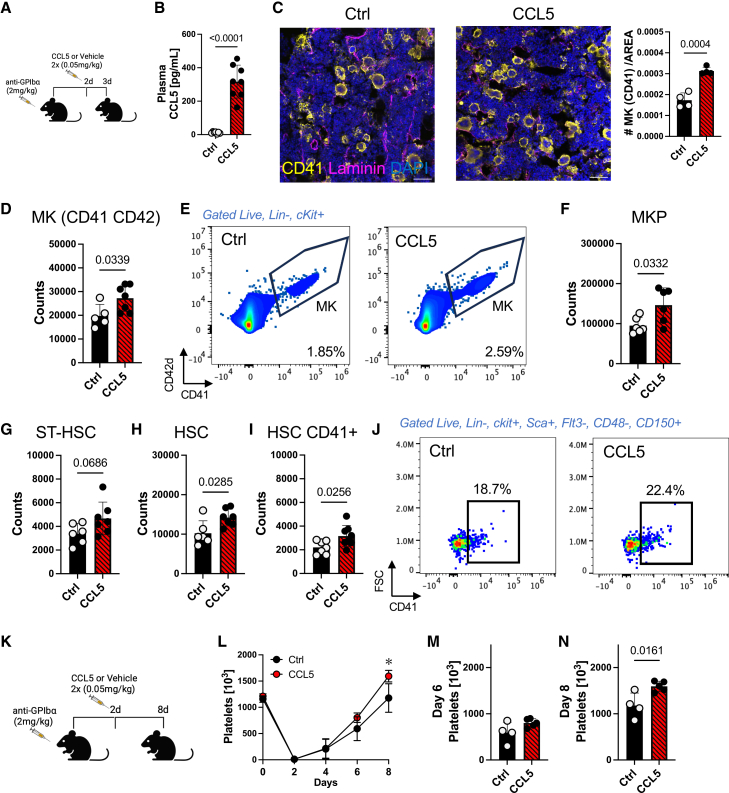
CCL5 facilitates platelet recovery follows anti-GPIbα antibody-induced platelet depletion (A) Schematic of antibody and CCL5 administration. Male mice were administered anti-GPIbα (2 mg/kg). After 2 days, mice were given CCL5 (0.05 mg/kg) or vehicle. 24 h post CCL5 administration, the bone marrow compartment was analyzed. (B) Quantification of CCL5 levels in plasma at 24 h by ELISA (*n* = 6–8). (C–E) Femoral cryosections from Ctrl and CCL5-treated mice were stained for CD41. (C) Representative bone marrow images showing MKs (CD41, gray). Scale bars, 200 μm. (D) Quantification of MKs (CD41^+^) per area using ImageJ (*n* = 4). (D) Quantification and (E) Representative plot of MKs (CD41^+^CD42^+^) by flow cytometry showing absolute counts (*n* = 5–7). (F–I) Absolute counts of (F) MKP, (G) ST-HSC, (H) HSC, and (I) HSC CD41^+^ quantified by flow cytometry (*n* = 6–8). (J) Representative flow cytometry plot shows CD41^+^ HSCs after CCL5 and vehicle administration. (K) Male mice were administered anti-GPIbα (2 mg/kg). After 2 days, mice were given CCL5 (0.05 mg/kg) or vehicle and monitored over 8 days. (L and N) Whole blood was drawn every 2 days using a tail nick. Platelets were measured using a hematology analyzer (Sysmex). (*n* = 4–5). Data are presented as mean ± SD. Statistical analyses were performed using an unpaired, two-tailed Student’s *t* test. *N* represents an individual mouse, and experiments were repeated 2–3 times.

To investigate the effects of CCL5 under thrombopoietic stress conditions, mice were treated with anti-GPIbα to induce platelet clearance with subsequent administration of either vehicle or CCL5 48 h later (Figure 4A). Twenty-four hours after CCL5 administration, we confirmed that there was elevated CCL5 in the plasma (Figure 4B). Correspondingly, we observed an expansion of MKs in the bone marrow of CCL5-treated mice (Figure 4C). Flow cytometry corroborated these findings, showing a significant increase in the frequency and absolute numbers of MKs compared to controls (Figures 4D and 4E). Additionally, MkP levels were significantly expanded (Figure 4F), aligning with observations under non-stress conditions (Figure 2A). Examination of HSPCs revealed a significant expansion of ST-HSC, HSC, and MK-biased HSC populations 24 h after CCL5 administration (Figures 4G–4J). Notably, while there was an increase in MPP2, no changes were detected in MPP3 (Figures S4A and S4B). Because CCL5 expanded immune-skewed MKs at homeostasis, we next assessed whether this phenotype extended to the stress setting. Following anti-GPIbα-induced thrombocytopenia, CCL5 treatment significantly increased the absolute number of MHC II^+^ MKs in the bone marrow compared with vehicle-treated controls (Figure S4C). Thus, in addition to expanding total MKs and MK progenitors, CCL5 augmented the immune-associated MK subset after platelet depletion.

As we saw a CCL5-mediated increase in MK counts following platelet depletion, we next aimed to assess if there was a functional impact on platelet recovery (Figure 4K). Platelet counts were measured every other day for 8 days (Figures 4L–4N). CCL5 treatment expedited platelet recovery, with significantly increased platelet counts on day 8 compared to vehicle-treated controls (Figures 4L–4N). Moreover, no significant differences were observed in other blood cell counts (Figures S4D and S4E). The increase in platelet counts reveals a functional downstream role for *in vivo* CCL5 treatment during stress hematopoiesis, namely, augmented platelet recovery after thrombocytopenia.

### CCL5 administration increases CCR1 and CCR5 receptor expression in MkPs and ST-HSCs

**Figure 5:**
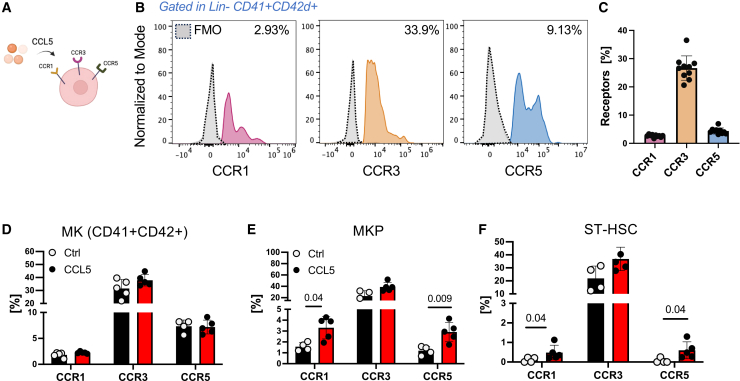
CCR1 and CCR5 are upregulated on MK progenitors (A) Schematic of CCL5 receptors CCR1, CCR3, and CCR5. (B and C) Representative histograms and quantification of CCR1, CCR3, and CCR5 expression in MKs by flow cytometry (*n* = 4–5). (D–F) Frequency of CCR1, CCR3, and CCR5 in (D) MKs, (E) MKPs, (F) ST-HSC (*n* = 4–5). Data are presented as mean ± SD. Statistical analyses were performed using an unpaired, two-tailed Student’s *t* test. *N* represents an individual mouse, and experiments were repeated 2 times. Illustrations were created with BioRender.

Previous studies from our group showed that CCL5 enhances MK maturation *in vitro* through the CCL5/CCR5 signaling axis (Machlus et al., 2016). To investigate this process *in vivo* and across different hematopoietic populations, we first analyzed surface expression of the main CCL5 receptors, CCR1, CCR3, and CCR5, on murine bone marrow MKs (Figure 5A). We confirmed that MKs express all three receptors on the membrane surface by flow cytometry (Figures 5B and 5C). Next, we investigated whether receptor expression patterns change in response to CCL5 stimulation after 24 h. While no differences were detected in mature MKs (Figure 5D), both CCR1 and CCR5 expression were increased in MkPs following *in vivo* CCL5 administration (Figure 5E). This was also observed in ST-HSCs, which exhibited elevated CCR1 and CCR5 expression (Figure 5F). This is consistent with the MK progenitor populations that were enhanced in response to CCL5 treatment.

### Megakaryocytes undergo metabolic reprogramming after CCL5 administration

**Figure 6:**
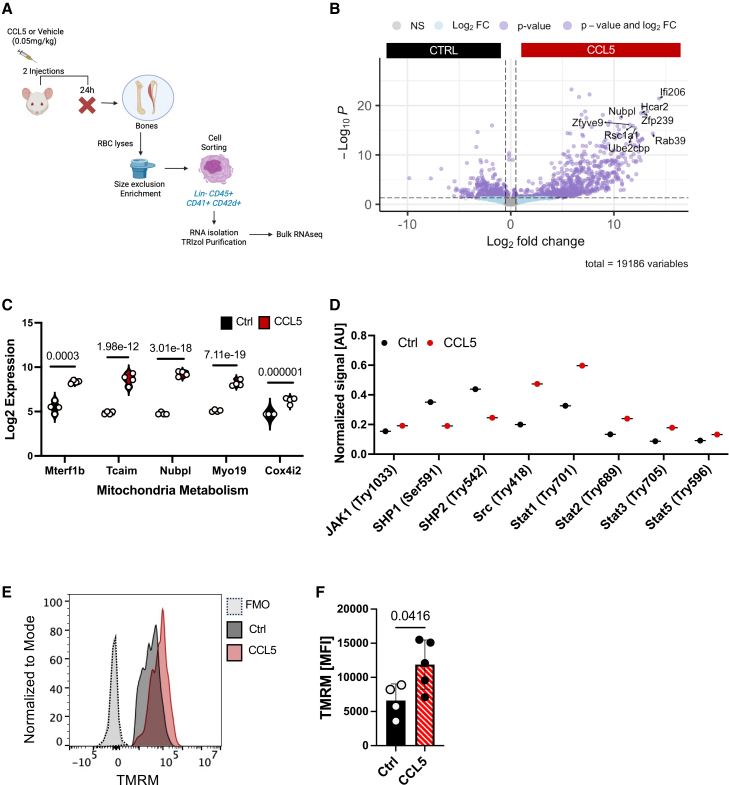
CCL5 administration induces transcriptional changes in megakaryocytes (A) Mice were treated with CCL5 as previously described, and bone marrow MKs were isolated after 24 h following lineage depletion and enrichment by flow cytometry sorting. RNA was extracted using TRIzol purification, and RNA sequencing was performed (4n vs. 4n). (B) Volcano plot shows genes that are significantly up- and down-regulated. The points highlighted in purple are genes that have padj <0.05 and a log2-fold change >1. Points in blue have a padj <0.05 and a log2-fold change <1. Gray points are non-significant. The dashed lines correspond to the cutoff values of log2 fold-change and padj chosen. (C) Differentially expressed genes associated with mitochondrial metabolism. Expression values are presented in log_2_ scale, and statistical significance was determined using padj. *N* represents a pool of 2 mice. (D) Male mice received retro-orbital injections of CCL5 (0.05 mg/kg) or vehicle (**Ctrl**). After 24 h, MKs were isolated by size exclusion, lysed, and analyzed using a RayBio Mouse JAK/STAT Pathway Phosphorylation Array. Signal intensity was quantified with Fiji. *N* represents a pool of 5 mice per group. (E) Representative histograms of TMRM in MKs by flow cytometry after CCL5 administration (Gray: FMO; Black: Ctrl; Red: CCL5). (F) MKs were identified by flow cytometry, and the mean fluorescence intensity of TMRM was quantified (*n* = 4–5). Data are presented as mean ± SD. Statistical analyses were performed using an unpaired, two-tailed Student’s *t* test. *N* represents an individual mouse. Illustrations were created with BioRender. *padj, adjusted p value.*

Together, our data reveal that CCL5 stimulates megakaryopoiesis in mice, resulting in an increase in MK progenitors and MK numbers (Figures 1 and 2). We next aimed to determine if MKs exhibited an altered transcriptome after CCL5 treatment. To assess the phenotypic changes occurring in MKs, we performed bulk RNA Sequencing (**RNA-Seq**) to evaluate the transcriptional impact of CCL5 administration. Mice were treated with CCL5 as previously described (Figure 1A), and bone marrow MKs were isolated after 24 h following enrichment via size-exclusion and cell sorting. RNA was extracted using TRIzol purification, and RNA sequencing was performed (Figure 6A). Bulk RNA-Seq analysis revealed 1,350 differentially expressed genes, with most transcripts upregulated in MKs isolated from the CCL5-treated group (Figures 6B, S5A, and S5B). Among the upregulated transcripts in the MKs from the mice treated with CCL5, we found genes related to immune response and inflammation (*Ifi206, Hrh4, Zfp697, Nlrp10*), mitochondria metabolism (*Mterf1b, Tcaim, Nubpl, Myo19, Cox4i2*), and cell cycle regulation (*Ccnjl, Nek3, Ptprv, Stox1, Ift88, Mlf1, Hdac11, Fbxw10*) (Figures 6C, S5C, and S5D).

To further probe signaling pathways that may be activated by phosphorylation, we isolated native MKs after 24-h CCL5 treatment and analyzed phosphorylation using a JAK/STAT phosphorylation array. We observed increased phosphorylation of JAK1, SRC, and STAT1-5 following CCL5 treatment, whereas phosphorylation of SHP1 and SHP2 was reduced (Figure 6D). These findings are consistent with previous studies showing that CCL5 can activate the JAK/STAT pathway, thereby promoting sustained inflammatory signaling (Jin et al., 2024; Wang et al., 2023). Together, these data indicate that the administration of CCL5 reprograms MK transcripts related to inflammation, mitochondria metabolism, and cell cycle, and further promotes the phosphorylation of JAK/STAT signaling proteins.

### CCL5 administration increases megakaryocyte mitochondrial activity

We next sought to characterize the functional outcome of the metabolic reprograming and activated signaling pathways downstream of CCL5 treatment. Several of the candidate genes are involved in mitochondrial metabolism (Figure 6C), and previous studies have linked CCL5-mediated pro-survival phenotypes to alterations in mitochondrial function (Mandal et al., 2011; Machlus et al., 2016). Further, STAT1, 3, and 5 (Figure 6D) can be found in mitochondria and contribute to the regulation of respiration, redox homeostasis, and mitochondrial transcription (Carbognin et al., 2016). Therefore, we measured differences in MK mitochondrial activity after 24-h CCL5 treatment. Specifically, we measured mitochondrial abundance, mitochondrial reactive oxygen species (**ROS**) levels, and mitochondrial membrane potential. No differences were observed in mitochondrial number or ROS levels by flow cytometry (Figures S5E and S5F). However, mitochondrial membrane potential was significantly increased after CCL5 administration, as assessed by TMRM fluorescence (Figures 6E and 6F). In summary, CCL5 treatment induced an inflammatory transcriptional program in MKs. Functionally, this was accompanied by the induction of JAK/STAT signaling and increased mitochondrial membrane potential, consistent with a model in which CCL5 enhances mitochondrial activity and supports MK proliferation and survival.

### CCL5 enhances megakaryopoiesis in a human iPSC-derived bone marrow organoid

**Figure 7:**
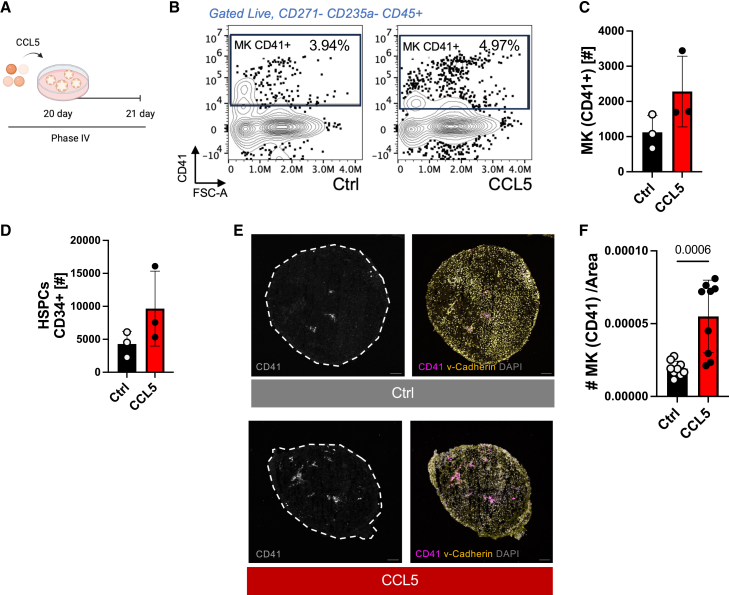
CCL5 promotes megakaryocyte expansion in human bone marrow organoids (A) At day 20 of organoid culture, organoids were treated with either vehicle or recombinant human CCL5 (50 ng/mL) for 24 h and 4 h prior to the end of the experiment. Organoids were processed for either. (B–D) flow cytometry by dissociation or (E–F) imaging after fixation and embedding. (B) Representative plot of MKs after vehicle and CCL5 administration. Absolute counts of (C) MKs and (D) HSPCs (CD34^+^). *N* represents technical replicates, and experiments were repeated 3 times. (E) Representative images of bone marrow organoid after vehicle and CCL5 administration showing MKs (CD41^+^, pink), vessels (v-cadherin, yellow), and nuclei (DAPI, gray). Scale bars, 100 μm. (F) Quantification of MKs (CD41^+^) per area using ImageJ (*n* = 9 organoids). *N* represents an independent organoid from 3 different experiments. Data are presented as mean ± SD. Statistical analyses were performed using an unpaired, two-tailed Student’s *t* test. *N* represents technical replicates, and experiments were repeated 3 times. Illustrations were created with BioRender.

We next aimed to validate key findings in a human system. We used human iPSC-derived BMOs to test the effect of CCL5 administration on megakaryopoiesis in a model myeloid niche. Organoids were cultured and differentiated as previously described (Olijnik et al., 2024; Khan et al., 2023). To mimic *in vivo* administration, CCL5 (50 ng/mL) was administered twice (24 and 4h prior to experimental endpoint) to mature BMOs (Figure 7A). This timing was selected to parallel the acute *in vivo* exposure window and capture early effects on MK output without prolonged remodeling of the organoid niche. We measured the number of MKs that differentiated per BMO via both flow cytometry and immunofluorescence microscopy. Flow cytometry revealed a tendency toward an increased number of CD41^+^ MKs (Figures 7B and 7C) and HSCs (CD34^+^) following CCL5 treatment (Figure 7D). Immunofluorescence revealed a significant increase in MK numbers in CCL5-treated BMOs compared with vehicle controls (Figures 7E and 7F), consistent with our murine data. Together, these findings demonstrate that CCL5 promotes megakaryopoiesis both *in vivo* in mice and in a physiologically relevant human BMO system, supporting a conserved effect on MK output. In this initial BMO analysis, we focused on overall MK and HSC output and did not assess immune MK surface markers or JAK/STAT signaling. More detailed immune phenotyping and signaling studies in BMOs will therefore be an important goal for future work to fully define CCL5 responses in human MK subsets.

## Discussion

Inflammation is a key component of innate immunity, essential for priming adaptive responses and orchestrating the effector phase. It can be triggered by trauma, injury, autoimmune reactions, infections, or even physiological conditions such as diet and exercise (Marques et al., 2013). Beyond its role in immune defense, inflammation profoundly impacts hematopoiesis, reprogramming hematopoietic cells and altering cell differentiation and function (Caiado et al., 2021; Pietras, 2017). Although both acute and chronic inflammatory states appear to reshape MK differentiation and phenotype, the direct effects of inflammatory cues on megakaryopoiesis are less well defined (Couldwell and Machlus, 2019). Among the soluble mediators driving these processes, chemokines such as CCL5 are particularly important (Marques et al., 2013).

Elevated levels of CCL5 have been reported in several inflammatory conditions, including atherosclerosis, inflammatory bowel disease, hepatic inflammation, and COVID-19 (Machlus et al., 2016; Couldwell and Machlus, 2019; Marques et al., 2013; Zeng et al., 2022). However, this increase is often part of a broader inflammatory context rather than an effect of CCL5 alone. To dissect the contribution of this chemokine, we adopted a reductionist approach, focusing specifically on its role in hematopoiesis with a focus on MK differentiation. A previous study from our group demonstrated that CCL5 enhances MK maturation and proplatelet formation via CCR5 using an *in vitro* fetal liver-derived MK system (Machlus et al., 2016). While this work established a role for CCL5 in MK biology, it did not address the *in vivo* bone marrow microenvironment or the contribution of other hematopoietic populations. The current study advances this work by adopting an *in vivo* approach focused on adult bone marrow megakaryopoiesis, enabling analysis of native MKs and progenitor populations, the resulting phenotype of MKs after CCL5 treatment, and the mechanisms that drive the downstream functional consequences.

Our data revealed that CCL5 administration acutely promotes megakaryopoiesis, resulting in a rapid increase in MK numbers within 24h in both murine models and a human iPSC-derived bone marrow organoid. Notably, these changes in MK and progenitor compartments translated into a clear functional outcome, as CCL5 treatment accelerated platelet recovery after anti-GPIbα-induced thrombocytopenia. These findings reveal that CCL5 may be a key component of an inflammatory response that contributes to an acute replenishment of the MK compartment. Mechanistically, our results suggest that this effect is mediated through CCR5 and/or CCR1 on MK progenitors, in line with recent studies demonstrating that CCL5 exhibits variable receptor affinity, binding preferentially to CCR5 and CCR1 with a lower affinity for CCR3 (Blanpain et al., 2001). Additionally, a recent study has shown that CCR5 marks a subset of HSCs that are myeloid-primed and expand with age (Yılmaz et al., 2026). Together, these data provide further insight into why CCL5 might transiently expand HSCs to differentiate into MKs.

Over the last 5 years, single-cell RNA sequencing studies have revealed transcriptional heterogeneity within the megakaryocytic lineage (Guo et al., 2024; Sun et al., 2021; Liu et al., 2021). However, the functionality corresponding to the heterogeneity within the MK population remains incompletely characterized. Among the subpopulations, “immune MKs” have been proposed to have a lower ploidy state and smaller size (Sun et al., 2021; Li et al., 2024). Further, immune MKs express genes related to leukocyte-mediated immunity, cellular responses to cytokines, and interferons (Sun et al., 2021; Liu et al., 2021). Notably, our data show that while CCL5 induces an increase in megakaryopoiesis, the resulting population of MKs is lower in ploidy and expresses MHC II and CD48 on the surface. Consistent with these observations at homeostasis, after anti-GPIbα-induced thrombocytopenia, we also observed an increase in MHC II^+^ MKs in CCL5-treated mice relative to controls. These data suggest that CCL5 biases the MK compartment toward an immune phenotype not only under basal conditions but also during thrombocytopenic stress, in parallel with accelerated platelet recovery. At the same time, the enhanced platelet recovery indicates that CCL5-treated mice generate sufficient platelet-producing MKs under stress conditions, whereas under homeostatic conditions, the expansion appears to favor immune-skewed MKs and does not result in a measurable increase in platelet counts.

MKs alter their transcriptome in response to inflammation (Couldwell and Machlus, 2019), acquiring a unique signature. Likewise, transcriptomic analysis of MKs isolated from CCL5-treated mice revealed upregulation in transcripts associated with cell cycle progression and proliferation, mitochondrial metabolism, and interleukin signaling. While the transcripts were isolated from MKs, it cannot be determined at which HSPC stage(s) CCL5 reprograms cell phenotypes. Recent studies have shown that acute inflammatory signaling can trigger cell cycle activation of a specific subset of HSCs, initiating the translation of MK transcripts and resulting in rapid maturation of MK progenitors (Haas et al., 2015). These findings align with our results demonstrating that CCL5 significantly increases proliferation in HSCs. A shift toward heightened metabolic activity and cell survival after CCL5 treatment may therefore promote proliferation cues and notably aligns with our previous observations of mitochondrial pro-survival signaling *in vitro* (Machlus et al., 2016).

Consistent with this transcriptomic profile, our analysis of native MKs conducted 24 h post CCL5 administration revealed increased phosphorylation of JAK/STAT signaling pathway proteins in response to CCL5. The CCL5/CCR5 axis is closely linked to JAK/STAT signaling and inflammatory responses; impairment of these pathways reduces CCR5 expression, indicating reciprocal regulation(Zeng et al., 2022). Activation of CCR5 by CCL5 can stimulate the STAT1 and NF-κB pathways, sustaining inflammatory signaling, while also activating JAK1/STAT3 and Src pathways that are involved in stress responses, cell migration, and survival signaling (Jin et al., 2024; Wang et al., 2023). In addition to the role of JAK/STAT signaling in inflammation, STATs can also be found in mitochondria.(Meier and Larner, 2014) A small fraction of STAT1, STAT3, and STAT5 (5%–10% of the total) localizes to mitochondria, functioning independently of cytoplasmic signaling and nuclear gene activation to regulate respiration and redox homeostasis (Meier and Larner, 2014). Mitochondrial STAT3, the best-studied, directly contributes to mitochondrial transcription and ATP production (Wegrzyn et al., 2009). Together, the transcriptomic and signaling data present a functional outcome of CCL5 administration. The culmination of these pathways then increases mitochondrial activation, which is a mechanism by which CCL5 may expand both MKs and MK progenitors.

This study has several limitations. First, our JAK/STAT and SRC phosphorylation data in MKs provide evidence of pathway engagement. However, additional genetic or pharmacologic inhibition studies *in vivo* will be important to address this more directly. In addition, because a variety of cells express and respond to CCL5, this chemokine is implicated in multiple biological processes (Zeng et al., 2022). Although we observed a specific effect on megakaryopoiesis at 24 h, we cannot fully exclude contributions from indirect effects on other CCL5-responsive cells within the bone marrow microenvironment. It is reassuring, however, that no changes were detected in red or white blood cells following CCL5 administration *in vivo* after 24, 48, and 96 h. Further, our previous publication laid the groundwork with *in vitro* studies that demonstrated CCL5-specific effects on MKs (Machlus et al., 2016). Finally, although we observed an increase in CD48^+^ MKs and MK progenitors, the precise differentiation pathway remains uncertain, as *in vivo* lineage-tracing experiments were beyond the scope of this study. Overall, these considerations suggest several avenues for future investigation but do not substantially alter our interpretation that CCL5 acutely promotes MK expansion, favors immune-skewed MKs, and supports platelet recovery *in vivo*.

In conclusion, we demonstrate that acute administration of CCL5 promoted a rapid and transient increase in megakaryopoiesis. In mice, 24h CCL5 exposure enhanced MK numbers in parallel with a transcriptomic shift that suggests changes in cell cycle progression, inflammatory signaling, and mitochondrial metabolism. This molecular reprogramming was supported functionally by the induction of JAK/STAT signaling and elevated mitochondrial membrane potential, consistent with heightened metabolic activity and pro-survival capacity. Notably, these effects were not limited to the murine system; CCL5 treatment of human iPSC-derived bone marrow organoids induced an acute increase in MKs, underscoring the conserved nature of this response across species. Together, these findings reveal that CCL5 promotes inflammation-driven hematopoietic adaptation by transiently expanding progenitor populations and enhancing MK counts. This acute boost in MK output may represent a mechanism to ensure timely MK adaptation during inflammatory stress, positioning CCL5 as a regulator of acute megakaryopoiesis.

## Resource availability

### Lead contact

Requests for further information and resources should be directed to the lead contact, Kellie R. Machlus (kellie.machlus@childrens.harvard.edu).

### Materials availability

Materials and protocols can be available to other investigators upon request.

### Data and code availability

RNA-seq raw data can be found in the repository SRA database: PRJNA135488.

## Acknowledgments

We acknowledge Dr. Khan for his contributions in establishing the organoid and supporting the process. This work was supported by the 10.13039/100000062National Institute of Diabetes and Digestive and Kidney Diseases (R03DK124746, R21DK142111, and R01DK139341 to KRM) and the 10.13039/100000050National Heart, Lung, and Blood Institute (R01HL151494 to KRM, K99HL175037 to MNB, and R35HL161175 to JEI) at the 10.13039/100000002National Institutes of Health, the 10.13039/100001422American Society of Hematology (ASH Restart Award to MNB, ASH Scholar Award to VC), the 10.13039/100000968American Heart Association (24IPA1274573 to KRM, 23POST1011433 to MNB, 25DIVSUP1476419 to VC), and 10.13039/100007299Harvard Catalyst Award to MNB.

## Author contributions

MNB designed and performed the experiments. V.C., I.C.B., J.T., and E.C. provided recommendations, performed the experiments, and interpreted data. K.G., D.H.L., D.F., E.M., S.B., B.Y., E.W., and O.M. performed the experiments. M.N.B. and K.R.M. conceptualized the project and wrote the manuscript. All the authors reviewed the manuscript. J.E.I. and K.R.M. funded the study. K.R.M. supervised the study.

## Declaration of interests

JEI has a financial interest in and is a founder of StellularBio, a biotechnology company focused on making donor-independent platelet-like cells at scale. JEI has a financial interest in and is a founder of SpryBio, a biotechnology company focused on using shelf-stable platelets to treat osteoarthritis. Boston Children’s Hospital manages the interests of JEI. All other authors declare no competing financial interests.

## STAR★Methods

### Key resources table

**Table:** 

REAGENT or RESOURCE	SOURCE	IDENTIFIER
**Antibodies**		

FITC-*anti*-P-selectin	Emfret Analytics	Cat#M130-1
PE-*anti*-activated integrin αIIbβ3	Emfret Analytics	Cat#M023-2
Anti-GPIbα antibody	Emfret Analytics	Cat#R300
BV785-*anti*-c-Kit	BioLegend	Cat#105841
FITC-*anti*-Sca-1	BioLegend	Cat#160908
BV711-*anti*-CD150	BioLegend	Cat#115941
Alexa Fluor 594-*anti*-CD105	BioLegend	Cat#120418
Alexa Fluor 700-*anti*-CD41	BioLegend	Cat#133926
APC-*anti*-CD42d	BioLegend	Cat#148506
PE-*anti*-CD16/32	BioLegend	Cat#101307
BV421-*anti*-CD135	BioLegend	Cat#135315
APC-Fire810-*anti*-Ter119	BioLegend	Cat#116264
PE-Cy7-*anti*-CD71	BioLegend	Cat#113812
BV605-*anti*-CD45	BioLegend	Cat#103140
PE-Cy5-*anti*-CD3	BioLegend	Cat#100274
PE-Cy5-*anti*-Gr-1	BioLegend	Cat#108410
PE-Cy5-*anti*-CD11b	BioLegend	Cat#101210
PE-Cy5-*anti*-NK1.1	BioLegend	Cat#156524
PE-Cy5-*anti*-CD19	BioLegend	Cat#115510
PE-Cy5-*anti*-Ter119	BioLegend	Cat#116210
PE-Cy5-*anti*-B220	BioLegend	Cat#103210
BV711-*anti*-Sca-1	BioLegend	Cat#108131
BV605-*anti*-CD150	BioLegend	Cat#115927
APC-Cy7-*anti*-CD48	BioLegend	Cat#103432
PE-*anti*-CD135	BioLegend	Cat#135306
FITC-*anti*-CCR1	BioLegend	Cat#152506
PE-Cy7-*anti*-CCR5	BioLegend	Cat#107018
BV421-*anti*-CCR3	BioLegend	Cat#144517
BV650-*anti*-MHC-II	BioLegend	Cat#107641
Pacific Blue anti-CD3	BioLegend	Cat#100214
Pacific Blue anti-Gr-1	BioLegend	Cat#108430
Pacific Blue anti-CD11b	BioLegend	Cat#101224
Pacific Blue anti-NK1.1	BioLegend	Cat#108722
Pacific Blue anti-CD19	BioLegend	Cat#152416
Pacific Blue anti-Ter119	BioLegend	Cat#116232
Pacific Blue anti-B220	BioLegend	Cat#103227
FITC-*anti*-CD3	BioLegend	Cat#100204
FITC-*anti*-Gr-1	BioLegend	Cat#108406
FITC-*anti*-CD11b	BioLegend	Cat#101206
FITC-*anti*-NK1.1	BioLegend	Cat#156508
FITC-*anti*-CD19	BioLegend	Cat#152404
FITC-*anti*-Ter119	BioLegend	Cat#116206
FITC-*anti*-B220	BioLegend	Cat#103206

**Chemicals, peptides, and recombinant proteins**		

Recombinant murine CCL5	Bio-Techne, R&D Systems	Cat#478-MR-025
ACK buffer	Gibco	Cat#A1049201
TRIzol Reagent	Invitrogen	Cat#15596026
Recombinant human CCL5	Bio-Techne, R&D Systems	Cat#278-RN-050
RNase A	Thermo Fisher Scientific	Cat#EN0531
Zombie Aqua	BioLegend	Cat#423102

**Critical commercial assays**		

BD Pharmingen BrdU Flow Kit	BD Bioscience	Cat#557892
DuoSet ELISA	R&D Systems	Cat#DY478
Proteome Profiler Mouse XL Cytokine Array	R&D Systems	Cat#ARY028

**Deposited data**		

RNA bulq analysis	This study	Cat#PRJNA1354881

**Experimental models: Cell lines**		

Gibco Human Episomal iPSC	Thermo Fisher Scientific	Cat#A18945

**Experimental models: Organisms/strains**		

C57BL/6J	Charles River Laboratories	Cat#027

### Experimental model and study participant details

#### Animal model

C57BL/6J mice were obtained from Charles River Laboratories. Male and females aged 7–10 weeks were used in the study. All animal protocols were approved by the Institutional Animal Care and Use Committee (IACUC) at Boston Children’s Hospital (protocol 0002148).

#### CCL5 injections

CCL5 injections were performed using recombinant murine CCL5 (478-MR-025, Bio-Techne, R&D Systems), with 0.1% BSA in PBS serving as a vehicle control. For the acute (24 h) treatment, CCL5 was administered retro-orbitally at 0.05 mg/kg twice: once 24 h and again 4 h before sacrifice (experimental endpoint). For the 48- and 96-h experiments, the same injection protocol was followed, and mice were euthanized 48 or 96 h after the first injection.

### Method details

#### Blood characterization

For non-terminal blood collection, mice were anesthetized with isoflurane, and 70 μL of blood was drawn from the retro-orbital capillary bed using heparinized capillaries and transferred into EDTA-coated tubes. Blood parameters, including platelet count and size, were analyzed using an automated cell counter (XN-1000, Sysmex).

#### Enzyme-linked immunosorbent assays and proteomic profiler

Cytokine levels in plasma and bone marrow fluid were determined using a mouse CCL5/RANTES DuoSet ELISA (DY478, R&D Systems) and a Proteome Profiler Mouse XL Cytokine Array (ARY028, R&D Systems). Plasma samples were diluted 1:2 in PBS, and bone marrow fluid samples were diluted 1:4 in PBS prior to analysis.

#### Platelet activation

Blood was collected as described above and diluted 1:100 in modified Tyrode’s buffer. Platelet agonists [adenosine-di-phosphate (ADP), collagen-related peptide (CRP), thrombin, and U46619] were added at the indicated concentrations to stimulate platelet activation. Samples were stained for 30 min in the dark with anti-P-selectin-FITC (M130-1, EMFRET) and PE-*anti*-activated integrin αIIbβ3 (M023-2, EMFRET). Platelet activation was quantified by mean fluorescence intensity (**MFI**) of P-selectin and activated integrin αIIbβ3 using a BD Accuri C6 Plus cytometer.

#### Megakaryocyte ploidy analysis

Bone marrow was isolated from a single tibia by centrifugation at 2,500×g for 40 s, as previously described (Heib et al., 2021). Cells were filtered through a 100 μm cell strainer, and red blood cells were lysed using ACK lysis buffer (Gibco, A1049201). After washing in PBS, cells were fixed and permeabilized in 70% ethanol/PBS for 30 min on ice. Samples were then treated with RNase A (Thermo Fisher Scientific, EN0531) and stained with anti-CD41-FITC antibody (BioLegend, 133904) and propidium iodide (Sigma-Aldrich, P1304-MP) for 30 min on ice. Ploidy distribution and the percentage of CD41^+^ cells were analyzed by flow cytometry using a BD Accuri C6 Plus cytometer.

#### Analysis of HSC and progenitor cells by flow cytometry

Bone marrow was isolated from one femur and one tibia by centrifugation at 2,500×g for 40 s, as previously described (Heib et al., 2021). Red blood cells were lysed in ACK buffer (A1049201, Gibco) for 5 min, and cells were passed through a 100 μm cell strainer. Cells were counted manually using a hemocytometer. To analyze the hematopoietic stem and progenitors, we used the following antibody panel: BV785-*anti*-c-Kit (105841, BioLegend), FITC-*anti*-Sca-1 (160908, BioLegend), BV711-*anti*-CD150 (115941, BioLegend), Alexa Fluor594-*anti*-CD105 (120418, BioLegend), Alexa Fluor700-*anti*-CD41 (133926, BioLegend), APC-*anti*-CD42d (148506, BioLegend), PE-*anti*-CD16/32 (101307, BioLegend), BV421-*anti*-CD135 (135315, BioLegend), APC-Fire810-*anti*-Ter119 (116264, BioLegend), PE-Cy7-*anti*-CD71 (113812, BioLegend), and BV605-*anti*-CD45 (148506, BioLegend), PE-Cy5-*anti*-lineage (anti-CD3 (100274, BioLegend), anti-Gr-1 (108410, BioLegend), anti-CD11b (101210, BioLegend), anti-Ter119 (116210, BioLegend), anti-B220 (1032120, BioLegend), anti-NK1.1 (156524, BioLegend), and anti-CD19 (115510, BioLegend) as described. Live cells were distinguished using Zombie Aqua (423102, BioLegend). HSPC populations were identified by spectral flow cytometry (Cytek Aurora) as previously described (Pietras et al., 2015; Pronk et al., 2007).

To analyze CCR1, CCR3, and CCR5 expression in cell populations, we used the following antibodies: BV785-*anti*-c-Kit (105841, BioLegend), BV711-*anti*-Sca-1 (108131, BioLegend), BV605-*anti*-CD150 (115927, BioLegend), APC-Cy7-*anti*-CD48 (103432, BioLegend), PE-*anti*-CD135 (135306, BioLegend), Alexa Fluor700-*anti*-CD41 (133926, BioLegend), APC-*anti*-CD42d (148506, BioLegend), FITC-*anti*-CCR1 (152506, BioLegend), PE-Cy7-*anti*-CCR5 (107018, BioLegend), BV421-*anti*-CCR3 (144517, BioLegend), BV650-*anti*-MHC-II (107641, BioLegend), Pacific Blue Lineage (anti-CD3 (100214, BioLegend), anti-Gr-1 (108430, BioLegend), anti-CD11b (101224, BioLegend), anti-NK1.1 (108722, BioLegend), anti-Ter119 (116232, BioLegend), anti-B220 (103227, BioLegend) and anti-CD19 (152416, BioLegend) as described. Live cells were distinguished using Zombie Aqua (423102, BioLegend).

#### Analysis of mitochondria-related parameters in MKs by flow cytometry

Mice were euthanized, and bone marrow was isolated as described above. Cells were stained using Alexa Fluor 700-*anti*-CD41 (133926, BioLegend), APC-*anti*-CD42d (148506, BioLegend), a PE-Cy5-*anti*-lineage panel (anti-CD3 (100274, BioLegend), anti-Gr-1 (108410, BioLegend), anti-CD11b (101210, BioLegend), anti-Ter119 (116210, BioLegend), anti-B220 (1032120, BioLegend), anti-NK1.1 (156524, BioLegend) and anti-CD19 (115510, BioLegend)), PerCp5.5-*anti*-CD45 (157612, BioLegend), Mito Green (M46750, Invitrogen), MitoSox Red (M36008, Invitrogen), TMRM (M20036, Invitrogen). Live cells were distinguished using Zombie Aqua (423102, BioLegend). MK population was identified as previously described by spectral flow cytometry (Cytek Aurora). Mitochondria-related parameters were quantified by assessing the MFI of 3,000 MK (Lin- CD41^+^ CD42d+) events.

#### Cryosectioning and immunofluorescence staining

Mice were euthanized and femurs were isolated and fixed in 4% paraformaldehyde (**PFA**) overnight. Femurs were transferred into a sucrose gradient (10%, 20%, and 30%) over 3 days. Femurs were sectioned at 10 μm using a CM3050 S Cryostat (Leica Biosystems) and transferred onto slides using a tape-transfer system (Kawamoto, 2003). Sections were rehydrated in PBS for 15 min, blocked using 5% goat serum, and stained using antibodies against CD41 (dilution 1:300; 133902, Biolegend) and laminin (dilution 1:400, L9393, Sigma-Aldrich) overnight. The next day, sections were incubated with secondary antibodies (dilution 1:500; goat anti-rat A488 and goat anti-rabbit A647, respectively). After washing in PBS containing 0.1% Triton X-100, DAPI was added for 5 min, and the slides were mounted using FluoroShield (Sigma-Aldrich). Image acquisition was performed using a 20x objective (Zeiss LSM880). MK number and area were quantified using Agilent BioTek Lionheart FX software.

#### BrdU assay

A total of 1 mg/mouse BrdU in 100 μL PBS was administered intraperitoneally 16 h before euthanasia. Mice were euthanized and BrdU incorporation in bone marrow cells was assessed using the BD Pharmingen BrdU Flow Kit (BD Biosciences) according to the manufacturer’s instructions. Bone marrow cells were isolated and first stained for surface markers as described in the section Analysis of hematopoietic stem and progenitor cells by flow cytometry. Cells were then fixed with Cytofix/Cytoperm buffer, permeabilized, and treated with DNase to expose incorporated BrdU. BrdU incorporation was detected using a fluorochrome-conjugated anti-BrdU antibody, and samples were analyzed by flow cytometry to determine BrdU incorporation on different bone marrow cell populations.

#### Platelet depletion assay

Mice were subjected to platelet depletion using an anti-GPIbα antibody administered at a dosage of 2 μg/g (R300, Emfret Analytics) via retro-orbital injection. A control group received a vehicle solution (PBS) following the same procedure. After a period of 48 h post antibody administration, CCL5 was injected as detailed in the Animals section. To monitor platelet recovery, tail nicks were performed every two days over an eight-day period, allowing for consistent assessment of platelet levels.

#### JAK/STAT phosphorylation array

A pool of native MKs (5 mice per condition) were isolated from by size-exclusion as previously described (Spindler et al., 2023) and lysed in RIPA buffer. Lysates from vehicle- and CCL5-treated native MKs were analyzed using the RayBio Mouse JAK/STAT Pathway Phosphorylation Array according to the manufacturer’s instructions. Briefly, membranes were blocked, incubated with samples, followed by a detection antibody cocktail and HRP-conjugated anti-IgG. Signals were developed using chemiluminescent detection buffers and imaged with a chemiluminescent imaging system. Array signal intensity was quantified using Fiji. Regions of interest (ROIs) were defined for each spot and measured integrated density. Background signal was determined from surrounding areas of the membrane and subtracted from each spot to obtain the corrected intensity. The normalized signal for each spot was calculated by dividing the corrected intensity by the average intensity of the positive control spots on the membrane for both control and CCL5 treatment.

#### MK sorting

Bone marrow was isolated from long bones of two mice per sample by centrifugation at 2,500 × g for 40 s. Cells were filtered through a 100 μm cell strainer, and red blood cells were lysed using ACK lysis buffer. MKs were isolated using a size-exclusion method (Spindler et al., 2023), followed by sorting via flow cytometry (Live, Lineage^−^, CD41^+^, CD42d^+^) using the following markers: FITC anti-Lineage (CD3 (100204, BioLegend), Ly-6G/Ly-6C (Gr-1) (108406, BioLegend), CD11b (101206, BioLegend), NK1.1 (156508, BioLegend), CD19 (152404, BioLegend), B220 (103206, BioLegend), Ter-119 (116206, Biolegend)) BV605 anti-CD45 (103139, BioLegend), APC Cy7 anti-CD41 (133928, BioLegend), PE anti-CD42d (148504, BioLegend), and Syto-40 (S11351, BioLegend). Live cells were distinguished using Zombie Aqua (423102, BioLegend). MKs were sorted using a 130 μm nozzle on a BD FACSymphony S6 Cell Sorter.

#### RNA isolation

Following isolation of MKs by sorting as previously described, cells were pelleted and lysed in 300 μL TRIzol Reagent (15596026, Invitrogen) and stored at −80°C. RNA was extracted according to the manufacturer’s protocol with minor adaptations. All procedures were performed under RNase-free conditions; benches were cleaned and pipettes treated with 100 mM NaOH or RNaseZap prior to use. Briefly, 200 μL chloroform was added per 1 mL TRIzol, the mixture was shaken vigorously for 15 s and incubated at room temperature (**RT**) for 2–3 min. Samples were centrifuged at 12,000 rpm for 15 min at 2°C-8°C to separate phases. The aqueous phase (∼60% of total volume) containing RNA was transferred to a new tube, and 500 μL isopropanol per 1 mL TRIzol was added. After a 15 min incubation at RT, samples were centrifuged at 12,000 rpm for 10 min at 2°C-8°C to pellet RNA. The RNA pellet was washed with 1 mL of 75% ethanol, followed by centrifugation at 7,500 rpm for 10 min at 2°C–8°C. The supernatant was removed, and pellets were air-dried before resuspension in RNase-free water. Purified RNA was stored at −80°C until use.

#### Bulk RNA sequencing and Bioinformatic analysis

RNA sequencing was performed at the Bauer Core at Harvard University.

*Instrumentation*: Libraries were prepared using the MANTIS Liquid Handler (Formulatrix) and the SciClone G3 NGSx workstation (Revvity). Full-length cDNA was prepared using the SMART-Seq v4 Ultra Low Input RNA Kit for Sequencing (Takara Bio USA, Inc.) and sequencing libraries prepared using the Nextera XT DNA library preparation kit (Illumina). *Methods*: Total RNA from each sample (4 vehicle vs. 4 CCL5-treated) was normalized between 1.8 ng prior to cDNA generation. The SMART-Seq v4 assay utilizes the SMART technology, switching mechanism at 5′ end of RNA template, to generate full-length cDNA using 13 cycles. The prepared cDNA was assessed for concentration using the Quant-iT Picogreen dsDNA assay kit (Invitrogen, P7589) on the SpectraMax i3 Multi-Mode Detection Platform (Molecular Devices) and normalized to 0.2 ng/μL prior to library prep. Full-length cDNA was fragmented using the Nextera technology in which DNA is simultaneously tagged and fragmented. Tagmented samples were enriched and indexed using 12 cycles of amplification with PCR primers, which included dual 8 bp index sequences to allow for multiplexing (Nextera XT Index Kit). Excess PCR reagents were removed through magnetic bead-based cleanup using PCRClean DX beads (Aline Biosciences) on a SciClone G3 NGSx workstation (Revvity). Resulting libraries were assessed using a 4200 TapeStation (Agilent Technologies) and quantified by qPCR (Roche Sequencing). Libraries were pooled and sequenced on one-half of an Illumina NovaSeq X Plus 10B lane using paired-end 150 bp reads.

*Bioinformatic analysis:* Samples were processed through the nf-core rnaseq pipeline version 3.14.0. (Ewels et al., 2020; Commnity tnc, 2025) Briefly, quality control of raw reads was performed using FastQC version 0.12.1 (Leggett et al., 2013) and adapter and quality trimming was performed using cutadapt version 3.4 and Trim Galore version 0.6.7(Marques et al., 2013). Trimmed reads were aligned to mouse genome GRCm38 using STAR version 2.7.9a [6] and quantified using Salmon version 1.10.1 (Dobin et al., 2013; Patro et al., 2017). Alignments were checked for evenness of coverage, rRNA content, genomic context of alignments (for example, alignments in known transcripts and introns), complexity, and other quality checks using a combination of FastQC, Qualimap version 2.3 (García-Alcalde et al., 2012), Samtools version 1.17 (Li et al., 2009), MultiQC version 1.19 (Ewels et al., 2016), and custom tools.

Differential expression by genotype was called at the gene level using DESeq2 version 1.38.3 (Love et al., 2014) using the counts per gene estimated by Salmon. Functional analysis of differentially expressed genes was examined for gene ontology (GO) terms, KEGG pathways, and Hallmark pathways using the ClusterProfiler R package version 4.6.2. Plots were generated using the R packages ggplot2 version 1.0.5, pheatmap version 1.0.12, enrichplot version 1.18.4, EnhancedVolcano_1.16.0 and ComplexHeatmap version 2.14.0. All of these analyses were performed using R version 4.2.3. Raw data can be found in the repository SRA database: PRJNA1354881.

#### Human iPSC-derived bone marrow organoid

Differentiation of Gibco Human Episomal iPSC (Thermo Fisher Scientific, cat. #A18945) into bone marrow organoids was performed as originally created and described by Khan et al. (Olijnik et al., 2024; Khan et al., 2023). At day 20, organoids were treated with either vehicle or recombinant human CCL5 (50 ng/mL; 278-RN-050, Bio-Techne, R&D Systems) 24 h and 4 h prior to the experimental endpoint. Detailed information on the differentiation protocol, organoid dissociation, flow cytometry analysis, organoid fixation, cryosectioning, and immunostaining procedures is provided later in discussion.

*iPSC Culture and Differentiation:* A Gibco Human Episomal iPSC (A18945, Thermo Fisher Scientific (discontinued) line was maintained in (A3349401, Thermo Fisher Scientific) and on Geltrex-coated 6-well plates (A1569601, Thermo Fisher Scientific). Cells were passaged as clumps using EDTA at 0.02% in PBS (0.5mM, E8008, Sigma) and were freshly thawed or passaged for differentiation and maintained in StemFlex supplemented with RevitaCell (A2644501, Thermo Fisher Scientific). Cultures were maintained at 37°C and 5% CO_2_. Differentiations were initiated with iPSCs between passages 5 and 30. Mycoplasma testing was performed during the experiment.

*Human iPSC-derived BMO:* Briefly, Phase −1 (aggregate generation): undifferentiated iPSCs were collected, dissociated, and seeded in ULA plates to form aggregates overnight. Phase I (mesodermal induction): aggregates were transferred into mesodermal commitment medium and cultured under hypoxic conditions until day 3 (D3). Phase II (vascular and hematopoietic commitment): aggregates were switched to hematopoietic and vascular commitment medium and transferred to normoxic conditions. Phase III (embedding in hydrogel): on day 5 (D5), aggregates were embedded in hydrogel to support sprout formation and cultured in hydrogel medium supplemented with cytokines. Organoids were re-fed on day 7 (D7) and day 10 (D10) with cytokine-supplemented medium, the latter with a reduced cytokine cocktail. Phase IV (collection and maturation): on day 13 (D13), organoids were extracted from the hydrogel, resuspended in maturation medium, and transferred into ULA 96-well plates. From day 16 (D16) onward, cultures were maintained with media changes every 72 h in maturation medium to support long-term differentiation. At day 20, organoids were treated with either vehicle or recombinant human CCL5 (50 ng/mL; 278-RN-050, Bio-Techne, R&D Systems) 24 h and 4 h prior to digestion. Following acute treatment (24 h), organoids were processed for either flow cytometry or imaging.

*Organoid Dissociation and Flow Cytometry:* Organoids were dissociated using collagenase/dispase (10 mg/mL; Sigma, 11097113001), prepared fresh before each experiment. Briefly, organoids (8 organoids pooled per technical sample) were collected using a pasteur pipette, ensuring no free-floating organoids were left behind. Organoids were pelleted at 400×g for 4 min, washed once with 10 mL PBS, and centrifuged again under the same conditions. The pellet was resuspended in 500 μL of collagenase/dispase and incubated at 37°C for 6 min in a tissue culture incubator. Following incubation, organoids were triturated with a P1000 pipette to disperse clusters, followed by a P200 once organoids had sufficiently fragmented. The digestion step was repeated with an additional 6 min incubation and trituration, continuing in 3 min intervals until a single-cell suspension was obtained. Enzymatic activity was stopped by adding PBS supplemented with 10% FBS, and cells were centrifuged at 400×g for 5 min. The resulting pellet typically contained two fractions, with smaller cells forming a halo surrounding a darker pellet of larger cells. Cells were counted using an automated cell counter (Countess II, Invitrogen). Single-cell suspensions were stained and analyzed on a Cytek Aurora flow cytometer using the following antibody panel: BV605-*anti*-CD41 (303742, BioLegend), FITC-*anti*-CD235a (349104, BioLegend), PerCP-*anti*-CD45 (304025, BioLegend), PE-*anti*-CD34 (343505, BioLegend), PE-cy7-*anti*-CD271 (345109, BioLegend), APC-cy7-*anti*-CD71 (334109, BioLegend), BV711-*anti*-CD31 (303135, BioLegend), and BV785-*anti*-CD11b (301345, BioLegend). Live cells were distinguished using Zombie Aqua (423102, BioLegend).

*Organoid fixation, OCT embedding, and Cryosectioning:* Organoids were transferred into 15 mL conical tubes using a pasteur pipette and allowed to settle by gravity (2–5 min). Medium was aspirated, leaving ∼0.5–1 mL, and organoids were washed once with PBS. Organoids were fixed in 3 mL of 4% PFA for 25–30 min at RT with gentle agitation. Following fixation, organoids were washed four times with PBS (5 min each, with gentle agitation) to remove residual PFA. Fixed organoids were stored in PBS at 4°C. For embedding, fixed organoids (8–12 per mold) were transferred into cryomolds, excess PBS was carefully removed, and organoids were covered with a thin layer of OCT (Tissue-Tek). Organoids were centered using a pipette tip, snap-frozen on dry ice in 100% ethanol, and the mold was then filled completely with OCT and returned to dry ice. Cryomolds were stored at −20°C (up to 6 months) or −80°C for long-term storage. Before sectioning, cryomolds were equilibrated to −20°C for 10–20 min. OCT blocks were mounted in the cryostat, oriented with the block in a diamond position relative to the blade and sectioned at 18 μm thickness. Sections were collected onto pre-warmed glass slides (three sections per slide) to improve adherence. Sections were optionally fixed in precooled acetone (0.815 M, −20°C, 20 min) and air-dried for 30 min at RT.

*Immunofluorescence staining of organoid sections:* Frozen organoid cryosections were equilibrated to RT for 20–30 min inside a humidified chamber. Sections were encircled with a hydrophobic barrier pen and rehydrated in PBS for 20 min, followed by a 5 min wash in PBS containing 0.1% Triton X-100. Sections were blocked in 5% donkey serum in PBS for 30–60 min at RT. Primary antibodies were diluted (typically 1:100) in blocking buffer and applied at 100 μL per section. Sections were incubated overnight at 4°C in a humidified chamber, followed by three washes in wash buffer (5 min each). Secondary antibodies (typically 1:300 in blocking buffer) were applied for 2 h at RT, followed by DAPI staining for 5 min. After three additional washes, sections were mounted with Fluoroshield. Slides were imaged within 24 h. Sections were imaged using a Zeiss LSM880 confocal microscope with a 40x objective and MK number and area were quantified using Fiji software (Version 2.14.0, NIH).

### Quantification and statistical analysis

#### Statistics

All results are presented as mean values with their corresponding standard deviations (SD). Statistical analyses were performed according to the specific tests outlined in the figure legends. For comparisons between two groups, unpaired, two-tailed Student’s t-tests were employed. For comparisons involving more than two groups, one-way or two-way analysis of variance (ANOVA) was used. Post-hoc tests, including Dunnett’s test for comparing each group with a control, as well as Sidak’s or Tukey’s corrections for multiple comparisons, were applied when appropriate to ensure robustness in the analysis of data variability. In all statistical tests, a *p*-value of less than 0.05 was considered indicative of statistical significance. The exact sample size (*N*) and its definition are specified in the corresponding figure legends.

## Supplemental information

Document S1. Figures S1–S6

Document S2. Article plus supplemental information
