# Supplementary material for: CCL5 exposure promotes acute and transient megakaryopoiesis and hematopoietic progenitor expansion
Source: Stem Cell Reports. 2026 Jul 16;21(8):103014. doi: 10.1016/j.stemcr.2026.103014 (PMC13476898; doi:10.1016/j.stemcr.2026.103014)
Supplement: Supplementary file 1 [file mmc1.pdf]

Stem Cell Reports, Volume 21

## Supplemental Information

# CCL5 exposure promotes acute and transient megakaryopoiesis and hematopoietic progenitor expansion

**Maria N. Barrachina, Dong H. Lee, Virginia Camacho, Karen G. Wang, Ethan Walsey, Estelle Carminita, Isabelle C. Becker, Siobhan Branfield, Daniela Freire, Emma Nikols, Emma L. Berdan, Blen Yohannes, Olivera Miladinovic, Julia Tilburg, Joseph E. Italiano, and Kellie R. Machlus**

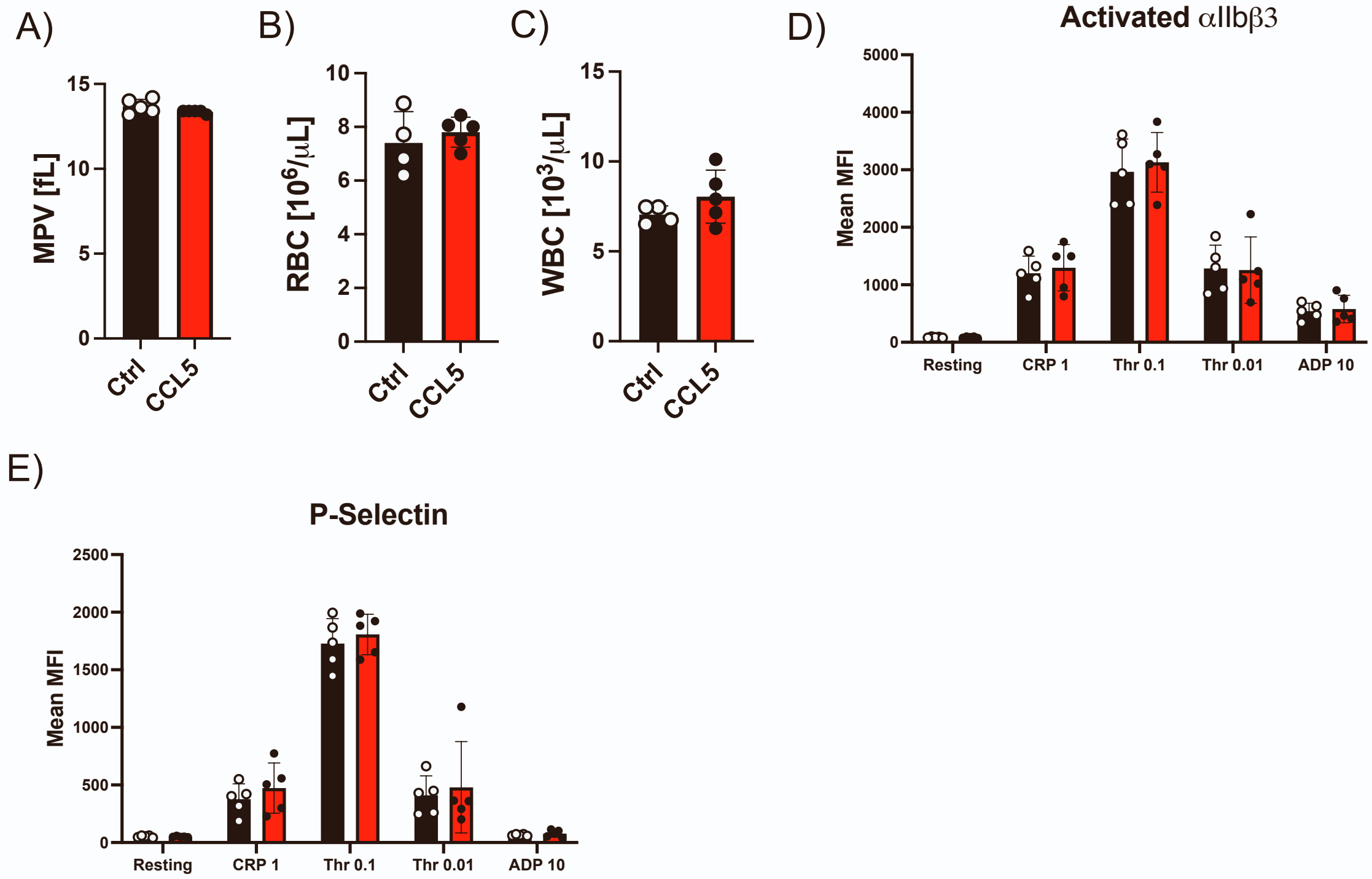


**Figure S1.** Complete blood counts were measured using an automated hematology analyzer, Sysmex, (A) MPV, (B) RBC, and (C) WBC. n = 4-5. (D) Platelet integrin αIIbβ3 activation (JON/A-PE) and (E) α-granule secretion (P-selectin-FITC) were determined ex vivo after stimulation of platelets from vehicle- or CCL5-treated mice with the indicated agonists at different concentrations (n = 5). Data are presented as mean ± SD. Statistical analyses were performed using an unpaired, two-tailed Student's t-test. N represents biological replicates, and experiments were repeated 2-3 times.

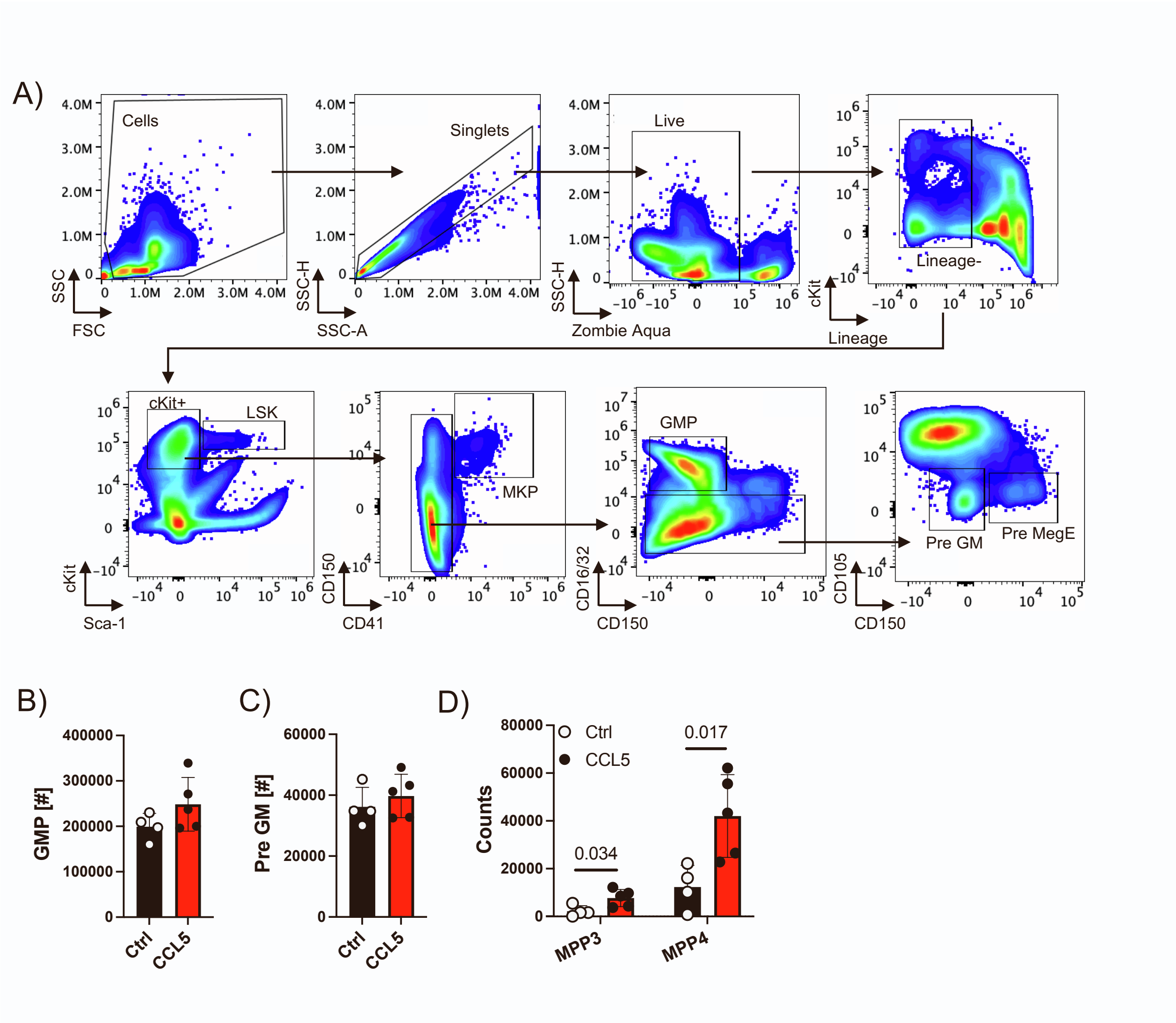


**Figure S2.** (A) Schematic of the gating strategy to identify megakaryocyte-erythrocyte and granulocyte-monocyte progenitors. (B-D) Absolute counts of (B) GMP, (C) Pre GM, and (D) MPP3 and MPP4 populations were quantified by flow cytometry. Data are presented as mean ± SD. Statistical analyses were performed using an unpaired, two-tailed Student's t-test. N represents biological replicates, and experiments were repeated 2–3 times. *PreGM, pre-granulocyte-monocyte; GMP, granulocyte-monocyte progenitor; MPP3, multipotent progenitor type 3; MPP4, multipotent progenitor type 4.*

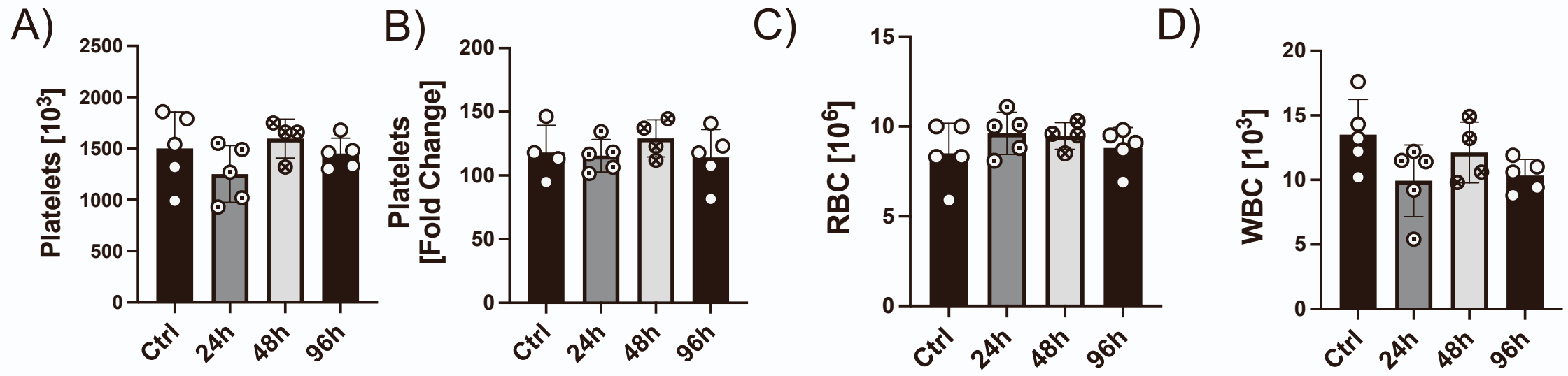


**Figure S3.** Complete blood counts were measured using an automated hematology analyzer, Sysmex. (A) platelets, (B) platelets fold change, (C) RBC, and (D) WBC counts. n = 4–5. Data are presented as mean ± SD. Statistical analyses were performed using one-way ANOVA with Dunnett's multiple comparisons test. N represents biological replicates; experiments were repeated 2 times.

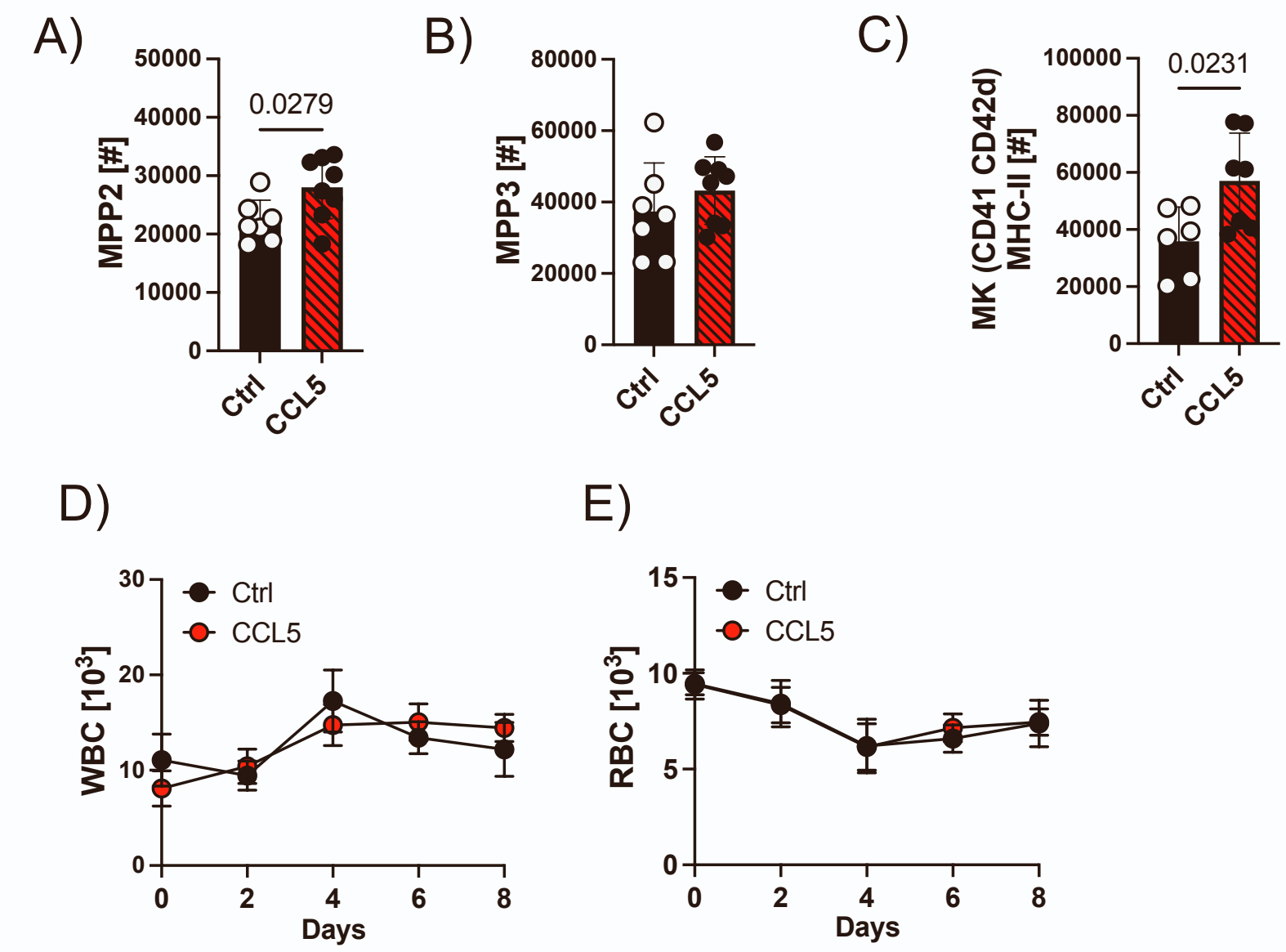


**Figure S4.** (A-B) Absolute counts of (A) MPP2, (B) MPP3 populations were quantified by flow cytometry. (C) Absolute counts of MKs expressing MHC II in murine bone marrow were quantified by flow cytometry. Data are presented as mean ± SD. Statistical analyses were performed using an unpaired, two-tailed Student's t-test. n = 6-8. (D-E) Complete blood counts were measured using an automated hematology analyzer, Sysmex. (E) RBC, and (F) WBC counts. n = 4-5.

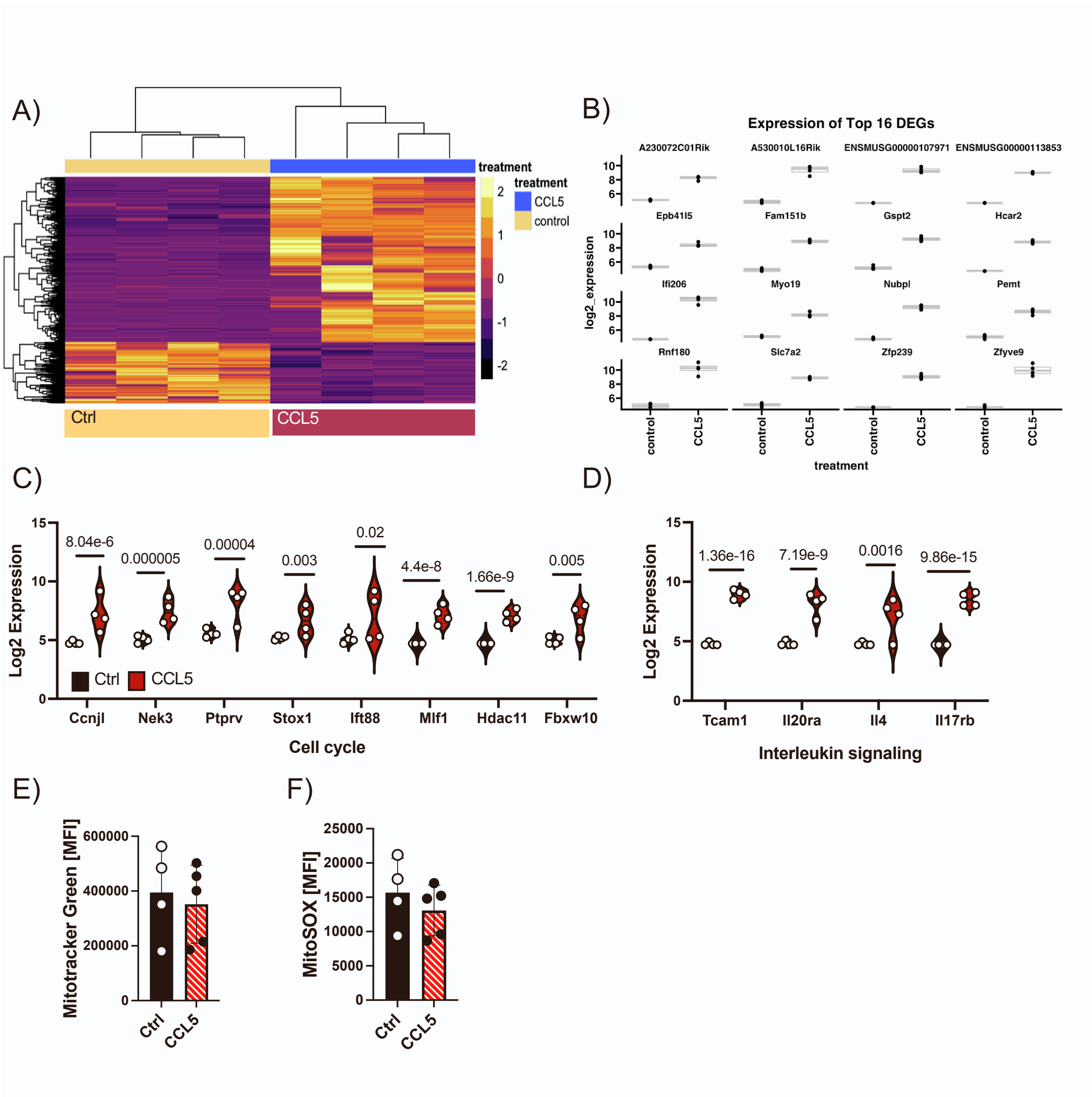


**Figure S5.** (A) Heat map showing differentially expressed genes related to vehicle- (yellow) and CCL5-treated (blue) groups. Expression values are presented as $\log_2$ fold change. Color scale represents relative expression levels (yellow= high, purple = low). Plots were generated using ComplexHeatmap version 2.14.0, in R (version 4.2.3). (B) Expression of the Top 16 differentially expressed genes. Expression values are presented in $\log_2$ scale. (C-D) Differentially expressed genes associated with (C) cell cycle and (D) interleukin signaling. Expression values are presented in $\log_2$ scale, and statistical significance was determined using padj. (E-F) Megakaryocytes were identified by flow cytometry and the mean fluorescence intensity of (E) Mitotracker green and (F) MitoSox was quantified (n = 4-5).

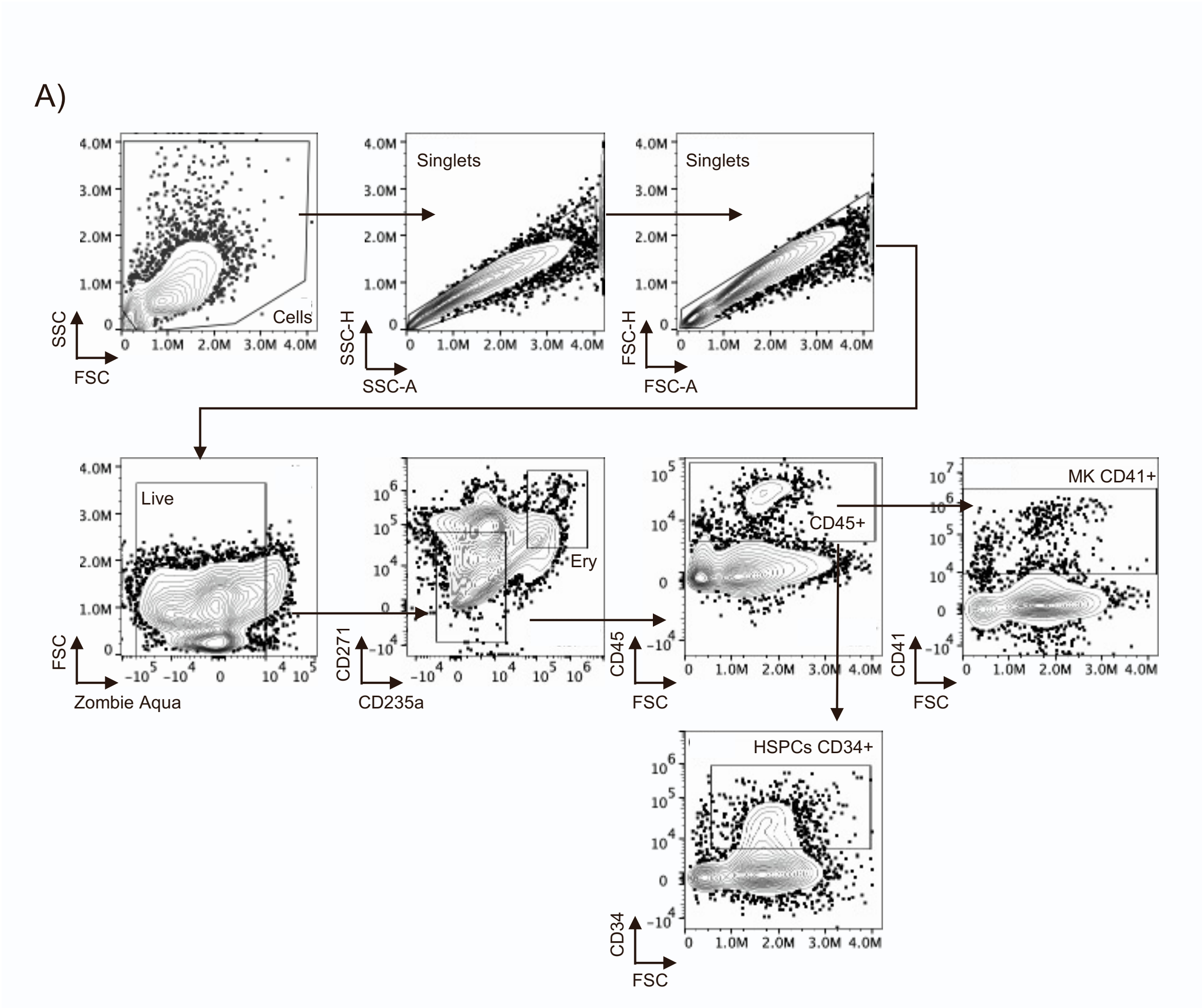


**Figure S6.** (A) Schematic of the gating strategy to identify MKs and HSPCs in the bone marrow organoid.
